# Ethical Considerations in Health Technology Assessment for Precision Medicine: A Delphi Study in a Greek Setting

**DOI:** 10.3390/jpm16060308

**Published:** 2026-06-05

**Authors:** Nikolaos Veskoukis, Nikos Stefanopoulos, Panagiota Naoum, Kostas Athanasakis

**Affiliations:** 1 Department of Nursing, University of Patras Campus, 26504 Rio, Greece; nvesk@yahoo.gr (N.V.); stefnik@upatras.gr (N.S.); 2 Laboratory for Health Technology Assessment (LabHTA), University of West Attica, Alexandras Av. 195, 11521 Athens, Greece; pnaoum@uniwa.gr

**Keywords:** health technology assessment, precision medicine, Delphi study, bioethics, equity, health disparities, genomic medicine, individualized care

## Abstract

**Background/Objectives:** Precision medicine has moved into routine practice, but its evaluation through Health Technology Assessment (HTA) remains ethically underdeveloped. Existing instruments do not address the distinctive ethical demands of genomic profiling, AI-based clinical decision-support, and the equitable distribution of benefits from high-cost targeted therapies. **Methods:** A modified two-round Delphi study was conducted with a multidisciplinary panel of 18 Greek experts in bioethics, HTA, genomic medicine, nursing, and health policy. In Round 1, 32 candidate ethical statements across seven thematic domains were rated on a three-point scale; retention required a Content Validity Ratio (CVR) ≥ 0.42 and ≥80% agreement. Retained statements were re-evaluated in Round 2 with consensus defined as median ≥ 2.0 and ≥80% agreement. Reporting follows ACCORD guidelines. **Results:** Fifteen of 32 statements satisfied retention criteria. In Round 2, all 15 achieved consensus with a median of 3.0 and agreement of 94.4–100% (interquartile range, IQR = 0.00). Five domains constituted the final framework: fundamental ethical principles; transparency, stakeholder participation, and institutional accountability; equity and access; digital health and artificial intelligence (AI); and pandemic preparedness and system resilience. Domains addressing environmental sustainability and social acceptability did not meet the threshold. **Conclusions:** This study presents, to our knowledge, one of the first empirically grounded ethical frameworks for precision medicine HTA developed within an EU Member State through a formal Delphi process. The framework is operationalised through a ready-to-use ethics checklist designed for direct integration into national HTA submission and appraisal processes. Conducted in Greece—a late-aligning EU Member State—the study provides a transferable methodological template for comparable health systems across Europe.

## 1. Introduction

Health Technology Assessment (HTA) supports reimbursement and coverage decisions on the basis of clinical effectiveness, cost, and an increasingly explicit set of ethical considerations [1,2]. As precision medicine has moved from research into routine practice, including increasing translation into primary care settings, the ethical demands placed on HTA have grown considerably [3,4]. The terms “precision medicine” and “personalised medicine” are used interchangeably in this manuscript, in line with current usage in European policy documents and in the IC2PerMed roadmap [5]. Precision medicine selects therapies on the basis of genetic, metabolic and environmental profiles. Its evidence base draws from small molecularly defined subgroups rather than large randomized trials, and AI-based decision support is now used across oncology, pharmacogenomics and rare disease diagnostics [6,7]. The unit cost of these technologies is high and concentrated, raising distributive questions that QALY-based cost–utility analysis is not designed to answer [8]. While some authors draw conceptual distinctions between the two terms, the present study follows the European Commission and IC2PerMed convention of treating them as functionally equivalent for the purposes of HTA policy development.

European HTA bodies including NICE, HAS and IQWiG have produced influential national frameworks [9,10], but methodological heterogeneity across Member States has persisted, including specialised domains such as rare disease and pediatric assessment [11]. Regulation (EU) 2021/2282 (HTAR), applicable from January 2025, introduced a common framework for Joint Clinical Assessments and Joint Scientific Consultations [10]. The HTAR builds on the EUnetHTA Core Model^®^ [9], which structures HTA across nine domains. The four clinical domains (clinical effectiveness, comparative clinical effectiveness, safety, and clinical effectiveness in subgroups) are subject to joint EU-level assessment, while the five non-clinical domains, including ethical, organisational, social and legal dimensions, remain a national responsibility under Article 13 [10]. The EUnetHTA Core Model contains a general ethics domain, but no validated framework calibrated to the specific normative challenges of precision medicine has been developed.

The gap is not confined to Europe. In the United States, the Institute for Clinical and Economic Review (ICER) acknowledged in 2023 the need to incorporate health equity into its methods [12], yet commercial health plans continue to apply HTA instruments without a precision medicine ethics framework [13]. In China, HTA has expanded considerably with a focus on drug reimbursement [14,15], but translating national precision medicine policies into accountable HTA practice remains unresolved [14,16]. Comparable challenges around ethical integration in HTA have been documented in the Eastern Mediterranean region, where assessments of ethical soundness in HTA reports remain methodologically heterogeneous [17]. The IC2PerMed initiative has called for ethically grounded governance of personalized medicine [5], yet has not been translated into operational HTA processes. A recent critical appraisal of ethical challenges in HTA confirmed that no empirically grounded ethical framework specifically designed for precision medicine assessment exists in any national or supranational HTA system [18]. Iran is not subject to EU regulatory requirements, and its HTA governance differs structurally from that of EU Member States. The reference is included because the methodological heterogeneity documented in the Eastern Mediterranean region reflects a pattern that is not regionally specific; the absence of structured ethics frameworks for precision medicine HTA is a cross-jurisdictional gap, not a European one. Including evidence from outside the EU strengthens the case for the framework developed here, by demonstrating that the problem the study addresses is global in scope even if the solution it offers is calibrated to a national context.

Greece offers a particularly instructive setting in which to address this gap. As one of the more recent adopters of formal HTA (introduced into reimbursement decision-making in 2018 [19]) and a Member State currently aligning its processes with the HTAR [10], it represents the kind of late-aligning health system for which practical methodological tools are most urgently needed. A recent five-year review of the Greek HTA process documents a system still maturing in procedural timelines and productivity [20], and Kyriopoulos et al. have set out the reform priorities now facing the system, including stronger governance structures and equity-focused interventions [21]. The ethical priorities that local stakeholders assign to precision medicine HTA may therefore differ from those identified in countries with longer-established traditions, with implications for national practice and broader European relevance. Against this backdrop, the present study identifies and prioritises ethical considerations for HTA in precision medicine in a Greek setting. The methodological and substantive implications of the findings, including their relationship to the HTAR mandate and the conditions under which the methodology can be transferred to other Member States, are developed in Section 4.6.

## 2. Materials and Methods

### 2.1. Study Design

A modified two-round Delphi methodology was employed to develop structured expert consensus on the ethical priorities relevant to health technology assessment in precision medicine. The Delphi technique is a well-established instrument in health policy and bioethics research for eliciting, refining, and aggregating expert judgement in domains where the empirical evidence base is limited, heterogeneous, or insufficiently operationalised to support direct synthesis [22,23,24]. Its iterative architecture, combining anonymous individual response with controlled group feedback, is particularly well suited to normative questions of the kind posed by this study, where divergence in expert opinion is itself informative and where the absence of a settled consensus is precisely the condition that motivates the inquiry.

A two-round structure was selected on methodological and practical grounds. The two rounds serve distinct and complementary functions: Round 1 performs content validation, establishing which candidate statements possess sufficient content validity to merit inclusion in a consensus framework; Round 2 performs confirmation of consensus: it establishes that retained statements command the degree of expert endorsement necessary to constitute a shared professional position. Given the three-point necessity scale employed, Round 2 does not produce a ranking or weighting of statements relative to one another; all retained statements that achieve consensus are treated as equally endorsed priorities within their respective domains. Separating content validity from confirmation of consensus in this way is consistent with best practice in Delphi research applied to the development of normative frameworks in health policy [23,24,25]. A two-round design also balances methodological rigour with panel retention, avoiding the attrition that longer iterative processes typically produce without sacrificing the structured feedback mechanisms that distinguish the Delphi approach from single-round surveys [25,26].

In Round 1, each candidate statement was rated on a three-point scale with response options of “necessary”, “useful but not necessary”, and “not necessary”. Content validity was assessed using the Content Validity Ratio (CVR) as proposed by Lawshe [27], calculated as CVR = (Ne − N/2)/(N/2), where N denotes the total number of panelists and Ne the number rating the item as “necessary”. Statements were retained for Round 2 if they simultaneously met a CVR threshold of ≥0.42 and achieved at least 80% agreement among panel members across the top two response categories. The use of a combined criterion, quantitative content validity alongside substantive expert endorsement, was intended to ensure that retained items were not merely statistically viable but reflective of genuine professional consensus on their importance to precision medicine HTA practice. CVR values range from −1.0 (all panelists rate an item as not necessary) to +1.0 (all panelists rate it as necessary). A value of 0.0 indicates that exactly half the panel endorsed necessity. For a panel of N = 18, the critical value at *p* = 0.05 (one-tailed) is CVR ≥ 0.42, following the revised critical values reported by Ayre and Scally [28], which corrected computational errors in Lawshe’s original tables. The one-tailed test is appropriate because the research question concerns whether the proportion of “necessary” ratings exceeds chance, not whether it differs from chance in either direction.

**Figure 1:**
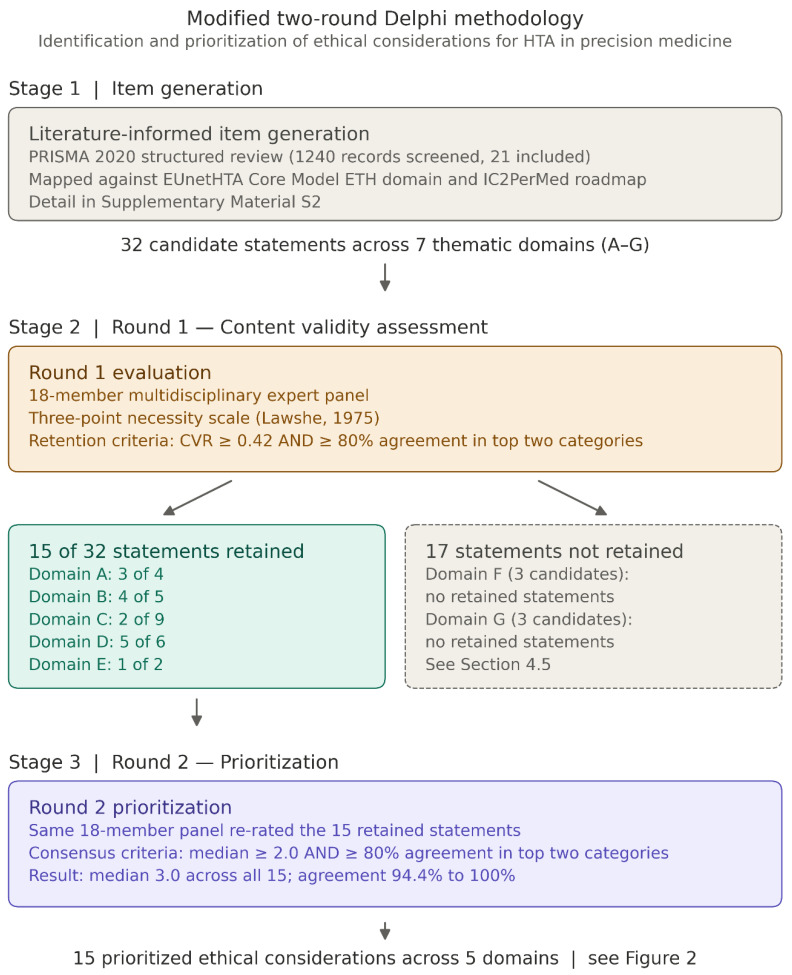
Modified two-round Delphi process for the identification and confirmation of ethical considerations for HTA in precision medicine. Stage 1: literature-informed item generation produced 32 candidate statements across seven thematic domains (A–G), supported by a PRISMA 2020 structured review (full documentation in Supplementary Material S2). Stage 2: Round 1 content validity assessment by 18 experts, with retention requiring CVR ≥ 0.42 and ≥80% agreement in the top two response categories; 15 statements were retained from 32 candidates: 3 of 4 from Domain A, 4 of 5 from Domain B, 2 of 9 from Domain C, 5 of 6 from Domain D, and 1 of 2 from Domain E. Domains F (3 candidates) and G (3 candidates) yielded no retained statements; methodological reasons are discussed in Section 4.5. Stage 3: Round 2 confirmation of consensus for the 15 retained statements by the same panel, with consensus defined as median ≥ 2.0 and ≥80% agreement in the top two categories; all 15 reached consensus (median 3.0, agreement 94.4–100%).

In Round 2, the 15 statements that satisfied these retention criteria were re-presented to the full panel for consensus confirmation using the same three-point scale. Consensus at this stage was operationalised as a median score of at least 2.0 and a minimum of 80% agreement across the top two response categories, thresholds applied consistently across both rounds to maintain comparability of the consensus standard throughout the process. The overall design is illustrated in Figure 1.

### 2.2. Questionnaire Development

Item generation followed a structured literature-informed approach. The research team conducted a structured systematic review of methodological guidance on the integration of ethics into HTA in accordance with PRISMA 2020, which screened 1240 records identified through PubMed, Embase, and Google Scholar for the period 2019–2024 and analysed 21 included studies addressing ethical analysis methodologies, emerging technology assessment, and equity considerations in HTA. From these sources, recurrent ethical themes were extracted and grouped to inform the construction of candidate statements. The full search strategy, screening process, PRISMA 2020 flow diagram, and list of included studies are provided in Supplementary Material S2. Item generation was further informed by a Delphi study on AI ethics and human rights [22], a Delphi study on optimising HTA for access to medicines [29], and a Web-Delphi on value aspects of medical devices [30]; by scoping and survey work on environmental sustainability in HTA [31,32]; and by recent proposals for HTA of AI-based technologies [33]. The derived items were then mapped against the ethics domain of the EUnetHTA Core Model^®^ [9] and the governance architecture of the IC2PerMed personalized medicine roadmap [5], ensuring that the candidate pool was anchored in the frameworks most directly operative within the European HTA context. This anchoring drew on both the empirical literature and the normative infrastructure that the study is designed to inform. It was intended to ensure content relevance while reducing the item generation bias that attends investigator-led questionnaire development when external normative reference points are absent.

Applying this approach yielded a pool of 32 candidate statements, organised across seven thematic domains. The seven-domain structure was constructed to test the consensus-readiness of the full breadth of ethical considerations identified in the source literature, including dimensions for which the methodological infrastructure of HTA is known to be less mature. Environmental sustainability and social acceptability were retained in the candidate pool on these grounds: not in expectation that they would necessarily achieve consensus, but to allow the panel to register substantively where consensus is and is not currently achievable. The interpretation of the resulting exclusions is developed in Section 4.5. Domain A covered fundamental ethical principles, encompassing justice, human dignity, patient autonomy, and the principles of non-maleficence and beneficence. Domain B addressed transparency, stakeholder participation, and institutional accountability. Domain C focused on equity and access, with particular attention to vulnerable and underserved populations. Domain D concerned digital health and artificial intelligence, addressing algorithmic bias, system explainability, human oversight, and the digital divide. Domain E covered pandemic preparedness and system resilience. Domain F addressed environmental sustainability and the ecological footprint of health technologies. Domain G examined social acceptability and public trust in health systems and their decision-making processes. Human dignity and patient autonomy are related but not coextensive. Autonomy is exercised by a subject who decides: it governs informed consent to genomic testing, the right not to know predictive results, and the conditions under which personal data may be shared. Dignity is not exercised—it is possessed. It constrains what may be done to a person regardless of their consent, their decision-making capacity, or the aggregate benefit to others. In precision medicine HTA, dignity becomes relevant precisely where autonomy cannot: in population-level allocation decisions, in the deployment of algorithmic systems that stratify patients without their individual knowledge, and in QALY-based thresholds that embed implicit valuations of life with disability or chronic illness. The two principles therefore generate non-overlapping assessment demands, which is why they appear as distinct criteria in the framework.

Statements were drafted to be self-contained and directly applicable to HTA practice, avoiding technical language that could introduce interpretation variability across the panel’s diverse disciplinary backgrounds. Each statement was rated on the three-point scale specified in Section 2.1. An open-ended item at the close of the instrument invited participants to raise additional considerations, suggest refinements, or flag perceived gaps in the thematic coverage.

The questionnaire was drafted and administered in Greek, the professional language of all panel members. The choice of Greek was methodologically deliberate rather than logistically convenient: where the research mandate is to establish a framework for national HTA practice, the normative judgements elicited from experts are necessarily shaped by their institutional experience, regulatory vocabulary, and professional culture, dimensions that are most accurately captured in the language through which that experience is habitually expressed and communicated [23,24].

The questionnaire underwent internal review by the research team for clarity, face validity, and comprehension prior to distribution. The review involved independent assessment by team members from different disciplines (bioethics, nursing science, and health policy), with feedback consolidated and incorporated before finalization. While this internal review is not equivalent to a formal external pilot study, it is a documented practice in focused Delphi studies where time, resource, or institutional constraints preclude broader pre-testing [29,34]. An estimated completion time of 10 to 15 min per respondent was confirmed during this process. The English-language statements presented in this manuscript and in Supplementary Table S1 were translated from the Greek originals by the bilingual research team and cross-checked for terminological consistency against the EUnetHTA Core Model^®^ and the IC2PerMed roadmap. Reporting of the full Delphi process follows the ACCORD guidelines for consensus methods in biomedicine [35]. Translation accuracy was secured through a structured two-stage procedure. Two bilingual members of the research team, one with primary expertise in bioethics and one in health policy, produced independent English renderings of each Greek statement without prior coordination. Divergences were resolved through structured dialogue until full terminological equivalence was confirmed. The agreed translations were then verified against both the EUnetHTA Core Model^®^ ETH domain terminology and the IC2PerMed governance vocabulary. This procedure follows the team-based adaptation of Brislin’s translation model recommended by Jones et al. for contexts where technical specificity and disciplinary diversity make individual translator equivalence insufficient [36].

### 2.3. Panel Selection

A purposive sampling strategy was employed to recruit a multidisciplinary panel of 18 experts, each with a minimum of five years of documented professional experience in at least one of the following fields: bioethics, health technology assessment, genomic medicine, nursing, or health policy. Purposive sampling was chosen to ensure that the panel reflected the full breadth of perspectives relevant to the ethical evaluation of precision medicine technologies within an HTA context. The decision to form the panel exclusively from Greek institutions reflects the regulatory context of the study: the HTAR explicitly assigns responsibility for the ethical, social, and organisational dimensions of HTA to national authorities, making a nationally grounded panel the methodologically appropriate instrument for this mandate [10]. At 18 members, the panel falls within the range typically recommended for focused Delphi studies in health policy and HTA research [23,24,25,37]. Recent methodological guidance on Delphi sample size confirms that panels in this range support valid content validity assessment when heterogeneity of expertise is prioritised over numerical scale [38,39]. Greece was selected as the national setting for three converging reasons. It is among the more recent adopters of formal HTA within the EU, having introduced structured reimbursement assessment only in 2018. It operates under the same HTAR mandate as all Member States, creating an immediate and documented need for national ethics frameworks that the joint EU-level clinical assessments will not produce. Its institutional and resource conditions are broadly representative of those facing other late-aligning Member States across Central, Eastern and Southern Europe, making the methodological template developed here transferable beyond the Greek context.

Panel composition was deliberately heterogeneous (Table 1). Bioethicists and legal scholars contributed expertise in public health law and research ethics. Health economists and HTA specialists brought experience in pharmaceutical policy and reimbursement decision-making. Nursing academics provided perspectives on health services organisation, quality of care, and patient safety. Researchers and clinicians working at the intersection of biomedical technology, computational health sciences, and precision medicine added technical and translational depth. Health policy experts with national and international advisory experience completed the institutional perspective. A patient advocate represented the perspective of civil society and organised patient communities. Together, these profiles provided the methodological, clinical, institutional, and experiential diversity that a rigorous Delphi process on this topic requires.

Participants were affiliated with leading Greek academic and research institutions, including the National and Kapodistrian University of Athens, the University of West Attica, the University of Patras, the University of Peloponnese, the University of Piraeus, and the National Technical University of Athens. Several panel members also held positions in national health bodies, including the National Agency for Quality Assurance in Health (ODIPY) and the National Organisation for the Provision of Health Services (EOPYY), bringing direct policy and governance experience to the panel.

Invitation was based on predefined inclusion criteria: documented involvement in research projects, institutional committees, or policy activities related to health technologies or ethical evaluation in healthcare. The same 18 experts participated in both rounds of the study, a design choice intended to preserve panel continuity and allow for meaningful controlled feedback between rounds.

The panel consisted of 13 females (72.2%) and 5 males (27.8%), with a median age of 49.5 years (range 37–62 years). All 18 invited experts completed both rounds of the study, yielding a panel retention rate of 100% across the full two-round process. Full retention reflects the relevance of the research topic to the professional community from which panelists were drawn, the relational groundwork established during the recruitment phase, and the purposive nature of the sampling strategy. Such retention eliminates the potential for differential attrition bias between rounds, a methodological concern that has been identified as a structural limitation in multi-round Delphi studies employing broader or more heterogeneous sampling strategies [23,24,25]. The implications of sampling choices for external validity are examined in Section 4.7.

### 2.4. Data Collection and Analysis

In Round 1, participants evaluated all 32 candidate statements using a structured questionnaire and the CVR-based retention criteria specified in Section 2.1. Statements meeting these criteria were carried forward to Round 2 (see Supplementary Table S1 for the full Round 1 content-validity results and retention outcomes).

Both Delphi rounds were conducted online between January and February 2026 via Google Forms (Google LLC, Mountain View, CA, USA). Participant anonymity was strictly maintained throughout both rounds to minimize bias and protect the integrity of responses. Between rounds, participants received controlled feedback comprising aggregated group results, including median ratings and percentage agreement for each statement, in line with the iterative design characteristic of the Delphi method.

Quantitative analysis was performed in Microsoft Excel (Microsoft Corporation, Redmond, WA, USA) and included descriptive statistics: medians to determine the central tendency of responses, percentage agreement to indicate the proportion of experts endorsing items in the top two response categories, and interquartile ranges (IQRs) to assess the degree of consensus or variability in expert ratings. Qualitative comments submitted alongside quantitative ratings were reviewed thematically [26] to contextualize and support the interpretation of expert opinion within each domain. Thematic analysis of Round 1 open-ended responses identified four recurring themes. Theme 1: Practical applicability: experts consistently noted the gap between normative acceptance of principles and their operational feasibility within current resource constraints. Theme 2: Professional training: multiple experts identified the absence of structured ethics training as a systemic barrier; this theme directly informed the retention of criterion B5. Theme 3: European harmonisation versus national contextualisation: experts acknowledged the HTAR mandate while emphasising that national conditions require context-specific adaptation. Theme 4: AI dynamics: the rapid pace of AI development was repeatedly cited as a reason to treat Domain D criteria as a living framework requiring periodic reassessment.

Qualitative coding was carried out independently by two members of the research team, and divergent interpretations were resolved through structured dialogue until consensus was achieved. The approach is consistent with the epistemological premises of thematic analysis, in which the goal is analytically grounded interpretation rather than mechanical replication of codes, and with the supplementary function of qualitative data in this study, where open-ended responses served to contextualize rather than determine the quantitative consensus findings [26,40].

### 2.5. Ethical Considerations

The study was conducted in accordance with the ethical principles of the Declaration of Helsinki [41] and received formal approval from the Research Ethics Committee, Department of Nursing, University of Patras (Protocol No. 96746, 7 November 2025). Before taking part in each round, all panel members were provided with a written information sheet setting out the study’s aims, the voluntary nature of participation, the anonymity of their responses, and their unconditional right to withdraw at any point without explanation or consequence. Electronic informed consent was obtained from each participant prior to the start of each round.

Participants responded through a structured questionnaire administered without identifiers; no personal or identifying data were collected at any stage of the Delphi process. The dataset was therefore fully anonymous from the point of collection, and no separate anonymization step was required prior to analysis. All data were stored securely on password-protected systems and processed in full compliance with Regulation (EU) 2016/679 of the European Parliament and of the Council (GDPR) [42].

## 3. Results

Of the 32 candidate statements submitted to Round 1 evaluation, 15 satisfied the pre-specified retention criteria and advanced to Round 2. The retention pattern across the seven candidate domains was uneven: three of four candidates originated from Domain A (fundamental ethical principles), four of five from Domain B (transparency, stakeholder participation, and institutional accountability), two of nine from Domain C (equity and access), five of six from Domain D (digital health and artificial intelligence), and one of two from Domain E (pandemic preparedness and system resilience). No statement from Domain F (environmental sustainability, three candidates) or Domain G (social acceptability and public trust, three candidates) met the combined retention threshold, and neither domain advanced to Round 2, a pattern that is itself methodologically significant and is examined in Section 4.5. Full CVR values and retention outcomes for all 32 statements are reported in Supplementary Table S1. Several excluded statements in Domains F and G achieved top-two agreement above 80%—notably F3 (94.4%) and G3 (94.4%)—but failed to meet the CVR threshold of ≥0.42. It was the content validity criterion, not the agreement criterion, that was not satisfied.

**Figure 2:**
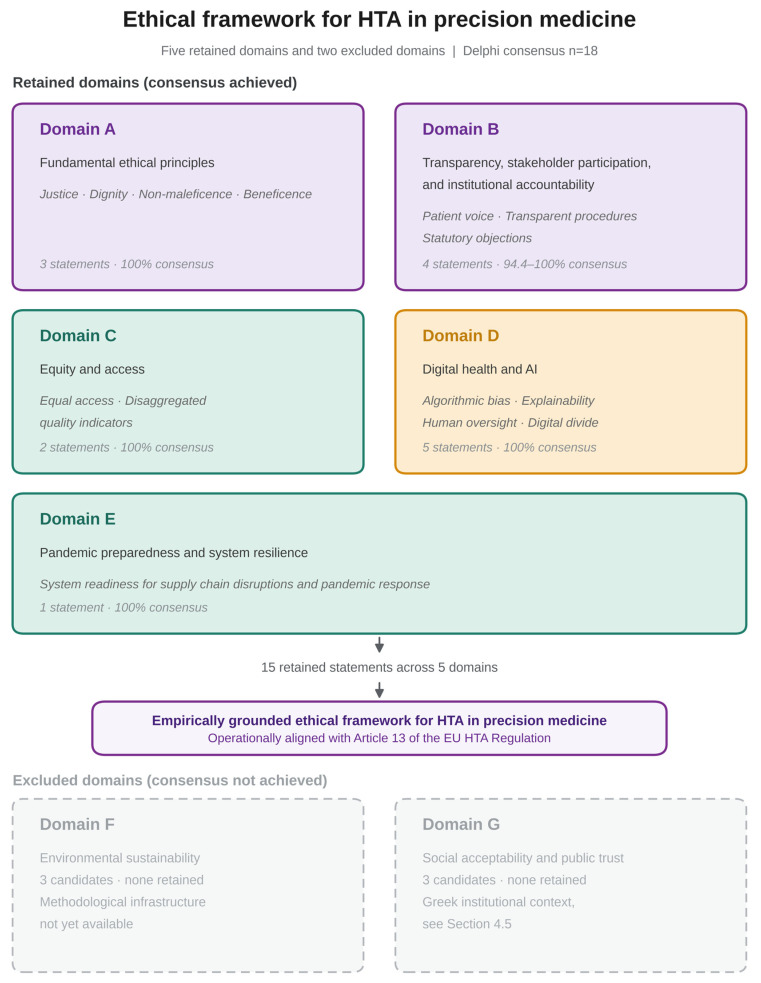
Ethical framework for HTA in precision medicine derived from a modified two-round Delphi study (N = 18 multidisciplinary experts, Greece). Five domains achieved consensus in Round 2 and constitute the final framework: fundamental ethical principles (A), transparency, stakeholder participation and institutional accountability (B), equity and access (C), digital health and artificial intelligence (D), and pandemic preparedness and system resilience (E), comprising 15 confirmed ethical statements. Two domains (F: environmental sustainability; G: social acceptability and public trust) did not meet the content validity threshold (CVR ≥ 0.42 and ≥80% agreement) in Round 1 and were excluded; the methodological reasons for these exclusions are discussed in Section 4.5. Colour coding groups thematically related domains: foundational principles (purple, A and B), distributive concerns (teal, C and E), and the technological frontier (amber, D); excluded domains are shown in dashed gray.

In Round 2, all 15 retained statements were re-submitted to the full panel for consensus confirmation using the same three-point scale. All 15 achieved the pre-specified consensus criteria, a median score of 3.0 and agreement in the top two response categories ranging from 94.4% to 100%, indicating a high and stable degree of expert consensus across all five thematic domains that advanced to this stage (Table 2). The interquartile range was 0.00 for all statements, with a median of 3.0 across the entire retained set, indicating that every statement was rated “necessary” by the majority of panelists. The five domains that constituted the final framework were fundamental ethical principles (Domain A), transparency, stakeholder participation, and institutional accountability (Domain B), equity and access (Domain C), digital health and artificial intelligence (Domain D), and pandemic preparedness and system resilience (Domain E), yielding a total of 15 prioritised ethical considerations for precision medicine HTA. The derived framework is illustrated in Figure 2. The uniform ceiling in Round 2 ratings is an expected methodological consequence of the strict content validity filter applied in Round 1: only statements with strong prior endorsement advance, and they are structurally predisposed to receive near-unanimous endorsement in a confirmatory round. The ceiling does not invalidate the consensus findings; it reflects the cumulative stringency of the two-stage design. Human dignity and patient autonomy appear as distinct Domain A criteria because they generate non-overlapping assessment demands, as developed in Section 2.2: dignity constrains population-level allocation decisions regardless of individual consent, while autonomy governs the conditions of individual participation in genomic medicine processes.

## 4. Discussion

This study set out to address a documented and consequential gap in the HTA literature: the absence of a structured ethical framework for precision medicine assessment calibrated to national HTA implementation. The Delphi process produced consensus across five of seven proposed thematic domains. Domains F (environmental sustainability) and G (social acceptability and public trust) did not retain any statement at content validation; the methodological significance of this exclusion is examined in Section 4.5. The 15 retained statements form the empirical core of the framework reported here. The sections below interpret the consensus thematically and situate it within the broader theoretical and policy context (Figure 2).

### 4.1. Beyond Utilitarianism: The Case for Ethical HTA

The consensus across Domains A and B carries a specific theoretical implication. Justice, dignity, autonomy, transparency and accountability did not enter the panel as competing priorities; they emerged as a coherent set, with no statement in either domain failing to reach consensus. This pattern fits less easily with the view that ethical considerations are a discrete dimension to be assessed alongside clinical and economic ones, and more easily with a position now articulated by the VALIDATE consortium and developed in recent qualitative work on HTA practice. On this view, facts and values in HTA are not separable, and methodological choices about evidence, comparators and outcomes are themselves normative choices [43,44,45]. HTA is not a technical exercise to which ethics is added; it is an inherently normative practice.

This implication runs against the dominant logic of HTA, which has remained largely utilitarian. Cost–utility analysis built around the QALY supports allocation under scarcity, but it aggregates welfare in ways that obscure distributional concerns and assigns implicit disvalue to life with disability or chronic illness [46]. Precision medicine sharpens these tensions. Evidence comes from molecularly defined subgroups rather than large randomized trials, and the questions raised concern genetic discrimination, the right to know one’s genomic profile, and the accountability of algorithmic decision-support [47]. None of these can be settled on aggregate utility grounds. The Round 1 data give this argument empirical purchase. Two candidate statements that articulated explicitly utilitarian or aggregate priority positions were among the most decisively rejected items in the entire pool: a statement framing utilitarian reasoning as the primary basis for HTA decisions (C7, CVR = −0.889) and a statement endorsing age-based priority allocation under resource constraints (C5, CVR = −0.778). The negative CVR values indicate that fewer than half of panelists rated these statements as necessary. The pattern of rejection differs across the two: nine of eighteen panelists rated C5 outright “not necessary”, while C7 attracted a different pattern of disapproval, with fourteen of eighteen panelists treating utilitarian primacy as “useful but not necessary” rather than as essential. Daniels and van der Wilt argued that, where principles cannot resolve disputes through utility reasoning alone, HTA must incorporate procedural justice and deliberative frameworks [48]. In 2023, ICER acted on this position, redesignating its “Contextual considerations” as “Special Ethical Priorities” and granting them institutional standing alongside the QALY [12]. The redesignation is informative because it came from within a framework whose evaluative architecture remains utility-centred.

Two institutional reference points sit closer to the present study and warrant a more careful comparison (Table 3). The EUnetHTA Core Model^®^ designates one of its nine domains for ethical analysis. The ETH domain organises ethical questions across six categories drawing on multiple methodological traditions [9]. As a structured question set it is effective. For precision medicine, however, it stops short of operational guidance. It does not say how privacy should be weighed against clinical utility in genomic testing, how genomic discrimination should be assessed, how equity should be operationalised in biomarker-based stratification, or what indicators a national HTA practitioner should apply. The ICER redesignation moves further in granting ethical considerations standing, yet retains utility maximization as its reference point and treats ethics as supplementary rather than constitutive [12]. Bloemen et al. argue that the normativity of HTA is expressed through ontological, moral and epistemological commitments that should be made explicit at each phase of an assessment [43]. The framework presented here is one attempt to do that for precision medicine in a national setting: empirically derived, organised by domain, and aligned with the responsibilities Article 13 of the HTAR assigns to Member States [10].

### 4.2. The Ethical Architecture of Precision Medicine HTA

Domains A (fundamental ethical principles) and B (transparency, stakeholder participation, and institutional accountability) achieved the most consistent consensus, with all retained statements receiving 100% agreement in Round 2 except statement B1 (94.4%). The pattern is informative. In precision medicine these principles carry direct operational consequences. Patient autonomy requires that genomic data collection is governed by consent processes that account for downstream use, including disclosure to family members or third parties. Non-maleficence engages the risk of genetic discrimination and the psychological burden of predictive testing for conditions without available intervention. Beneficence becomes inseparable from equity: a therapy that reaches only those who can afford it, or those whose genetic profiles are well-represented in training datasets, delivers selective good [49,50].

Patients and professionals do not always weigh these principles in the same way. A mixed-methods synthesis found that patients prioritised autonomy and non-maleficence, while professionals emphasised beneficence and systemic efficiency [49]. A systematic review confirmed that patients place particular emphasis on privacy, data security and genetic discrimination, concerns often underweighted in provider-led assessments [50]. European public surveys report high willingness to participate in genomic initiatives accompanied by sustained concerns about data governance and informed choice [51]. Consensus on Domain B principles is therefore not a procedural ornament. Without transparent processes that admit patient voice, the principles of Domain A risk being declared rather than enacted.

A methodological caveat applies to the consensus on Domain B. Patient representation on the panel was limited to a single advocate. Notably, statement B1 was the only retained statement across Domains A and B that did not reach unanimous Round 2 consensus (94.4%, indicating a single dissenting voice in a panel of 18). The arithmetic does not allow attribution of that dissent to any specific panelist. The pattern is nonetheless consistent with the broader literature: a patient-led consensus process would likely have produced different relative weightings, particularly on transparency and stakeholder participation criteria where patient and professional priorities are documented to diverge [49,50]. Arcà et al. document that structural barriers to patient engagement persist under the HTAR, with procedural guidance on patient involvement in joint scientific consultations remaining underdeveloped [52]. Wale et al. report that patient involvement in HTA consensus work remains low across European settings [53]. The limited patient representation on the present panel is therefore not a methodological flaw specific to this study but a structural feature of HTA consensus work documented across the international literature [52,53,54]. The Domain B priorities should be read as a provisional foundation, to be tested through patient-led consensus work as those methodologies reach operational maturity [30,52,53,55].

The substantive content of Domain B has been developed elsewhere in ways that bear on the present findings. Peacocke et al. note that topic selection in HTA is itself a normative act, reflecting implicit value judgements about what matters [56]. HTAi and ISPOR good-practice guidance for HTA guideline development calls for explicit articulation of the values underpinning methodological choices [57]. Hoxhaj et al. show that ethics and HTA training among healthcare professionals across Europe remains limited and heterogeneous, indicating a structural capacity gap that institutional accountability alone cannot close [58]. Domain B addresses the conditions under which Domain A operates. Domain C, examined next, raises a different question: who has access to the technologies that pass assessment.

### 4.3. Justice, Access, and System Resilience

Domain C (equity and access) and Domain E (pandemic preparedness and system resilience) sit together because both raise the same underlying question: who has access to precision medicine, and under what conditions [59]? Domain C produced consensus on two retained statements: equal access to health services as an evaluative criterion (C1) and disaggregation of health quality indicators by social group (C4). The pattern across the wider Domain C candidate pool is itself informative. Statements naming vulnerable populations (C2: migrants, persons with disabilities, low-income groups) and proposing cost ceilings on orphan therapies (C6) did not achieve content validity, with C6 attracting the most decisive rejection in the entire pool (CVR = −0.889). The retained statements thus frame equity as a procedural and evidentiary commitment, not as a substantive allocation rule that names which groups receive priority or which technologies are excluded on cost grounds. The cost structure of precision medicine is not neutral with respect to this question. Development costs are high, indications are narrow, and research investment is concentrated in commercially attractive genetic profiles. The result is access barriers that fall disproportionately on lower-income populations and underrepresented genetic communities [8,60]. The decisive rejection of C6 is not an isolated finding. It is consistent with the position of the European Economic and Social Committee in its February 2026 opinion on the European Life Science Strategy and Research and Technology Infrastructures (TEN/867) [61], which cautions that rigid cost-effectiveness thresholds structurally disadvantage small patient populations and substitute a distributional mechanism for what should be a deliberative one. The panel endorsed equity as a procedural commitment and rejected equity enforced through cost restrictions—the distinction between equity as process and equity as gatekeeping that the EESC draws, now empirically grounded in Greek expert consensus.

Without structured equity assessment within HTA, precision medicine can widen disparities rather than narrow them. Khoury et al. argue that there is a closing window in which to address implementation inequities before they become entrenched [62]. A 2025 WHO analysis of human genomics in clinical research over more than three decades confirms that populations outside Europe and Asia remain severely underrepresented in genome-wide association studies [63]. AI systems trained on these datasets are structurally ill-equipped to deliver equitable benefits across the full diversity of human populations. The equity criterion of Domain C is therefore a structural safeguard, not a procedural refinement.

The Greek context gives this concern empirical weight. The healthcare system entered the precision medicine era with unmet needs already increased and inequalities widened along income and geographic lines after a decade of austerity [64,65], and these vulnerabilities persist in current data on rural–urban access disparities [21]. For a system in which equity is at once stated priority and ongoing challenge, including it as a core HTA criterion is not optional; it is constitutive.

Domain E reaches the same distributive question from a different angle. The COVID-19 pandemic showed that equitable access to essential technologies during a crisis depends on supply chain governance and international solidarity arrangements that are themselves matters of distributive justice [66,67,68]. Haldane et al. found that countries with stronger pre-pandemic resilience achieved more equitable outcomes across socioeconomic groups, indicating that resilience is a precondition for justice rather than a separate technical property [68]. The WHO Pandemic Agreement of 2025 has now embedded this recognition in international governance [69]. That a Greek expert panel reached consensus on pandemic preparedness as an ethical priority for precision medicine HTA reflects both local experience of system fragility and a wider global imperative.

### 4.4. The Technological Frontier: AI, Data, and the Ethics of Algorithmic Medicine

Domain D produced the most decisive consensus of the study: five of six candidate statements were retained at content validation. The retained statements clustered around four substantive priorities: detection and mitigation of algorithmic bias (D2), explainability of AI-based clinical decision systems (D3), meaningful human oversight (D4), and the digital divide (D5). Statement D1, which holds that ethical evaluation should apply to digital health technologies as such, functions as the framing principle under which the four operational priorities sit. These priorities show thematic alignment with three independent international initiatives. The decisive consensus in Domain D reflects two convergent factors. The first is regulatory saturation: by the time this study was conducted, the EU AI Act had classified medical AI systems as high-risk and mandated transparency, human oversight, and post-market monitoring, while FUTURE-AI and STANDING Together had produced international consensus on bias monitoring and explainability. Panel members encountered the Domain D criteria not as novel proposals but as already-codified professional obligations. The second factor is experiential proximity: Greek clinical communities have engaged directly with algorithmic applications in oncology and pharmacogenomics, making the operational stakes of bias and opacity directly clinical rather than theoretical. The combination produced a domain where the expert community did not need to be convinced of the normative case, only asked to confirm it.

STANDING Together (350+ researchers, 58 countries) identified bias monitoring and transparency in health data sharing as the most critical prerequisites for trustworthy AI in healthcare [70]. FUTURE-AI (117 experts, 50 countries) reached consensus that explainability and human oversight are central to deployable AI in clinical settings [71,72]. The EU Artificial Intelligence Act (Regulation 2024/1689) [73], applicable to high-risk AI systems including those used in medical decision-making, mandates transparency under Article 13, human oversight under Article 14, and post-market monitoring under Article 72. Each of these maps onto the present panel’s priorities.

The alignment requires careful reading. Panel members were drawn from academic and policy environments in which the FUTURE-AI and STANDING Together frameworks were already known and discussed. Full independence between the consensus processes cannot be claimed. What the alignment indicates is that the priorities identified for AI-driven precision medicine HTA are not idiosyncratic to the Greek context. Jobin et al. report substantive convergence across 84 global AI ethics guidelines on transparency, justice, non-maleficence and accountability, while flagging the continued need for context-specific interpretations at the national level [74]. The convergence reported here strengthens, without replacing, the case for cross-national replication.

Convergence also has methodological consequences. AI ethics literature has been criticized for producing principles without operationalisation, broad commitment to fairness and transparency that gives little practical guidance to HTA practitioners [75,76]. The Domain D findings are anchored in the specific context of precision medicine assessment. They concern AI systems used to support genomic interpretation, treatment selection and risk stratification. The stakes of opacity and inadequate oversight in these applications are directly clinical. A systematic review in the Annals of Internal Medicine documents consistent associations between healthcare algorithm use and the widening of racial and ethnic health disparities across multiple clinical domains [77]. Di Bidino et al. have proposed an HTA framework for AI-based technologies that addresses several of these dimensions but note that its implementation requires explicit normative guidance of the kind this study provides [33].

### 4.5. The Limits of Assessment: Environmental Sustainability and Social Acceptability

Two domains did not yield retained statements following Round 1 content validation: environmental sustainability (Domain F) and social acceptability and public trust (Domain G). No statement from either domain met the combined CVR threshold (≥0.42) and 80% agreement criterion. The pattern is informative. It reflects the immaturity of the evidence base in these areas and the structural features of the Greek institutional context.

Domain F, which addressed the ecological footprint of precision medicine technologies, including the energy demands of large-scale genomic data infrastructure, did not achieve content validity despite strong individual interest from several panelists. This is consistent with findings from Pinho-Gomes et al., whose scoping review found that environmental sustainability remains at the margins of HTA and clinical guideline development globally [31], as well as Bobini et al., who documented that HTA stakeholders internationally acknowledge the importance of environmental considerations but lack validated methods for their incorporation [32]. The European Sustainable HTA Working Group, established in May 2024, is developing such methods [78], but they do not yet exist in a form applicable to routine assessment. The absence of retained statements in Domain F reflects not disagreement about values but the absence of methodological infrastructure for translating those values into assessable practice. Beyond this global methodological gap, the exclusion of Domain F also reflects a deliberate prioritisation. In a system consolidating its core HTA capacity [20,21], the panel converged on ethical dimensions whose operational instruments are currently mature and immediately applicable to assessment practice. This pattern is consistent with the broader European trajectory, in which sustainability considerations are being progressively integrated into HTA as the methodological tools mature [78].

Domain G presents a different and more instructive pattern. Social acceptability covers public trust in health systems, the perceived legitimacy of HTA decisions, and the cultural dimensions of technology adoption. The Greek context introduces complexity here. The OECD survey on trust in public institutions found that only approximately 30% of Greek citizens express satisfaction with the national healthcare system, against an OECD average of 52% [79]. Souliotis et al. have shown that roughly half of Greek citizens view health system reforms negatively even when they acknowledge their necessity, a paradox that reflects deep structural ambivalence toward institutions [80]. Ervasti et al. traced this ambivalence to longer-standing features of Greek society that predate the austerity period, situating low institutional trust as a relatively stable structural characteristic rather than a crisis-specific reaction [81]. Yet Ladi et al. showed that trust is recoverable through transparent, evidence-based policymaking, and that the Greek response to COVID-19 offered tentative evidence of this [82]. The failure of Domain G statements to achieve content validity signals not that social acceptability is unimportant, but that its operationalisation within HTA requires context-sensitive instruments and validated measurement frameworks that the field has not yet developed.

The exclusions also admit a substantive reading. The panel may have judged that environmental sustainability and social acceptability, however important in broader health policy debates, are not yet ripe for inclusion as formal HTA criteria for precision medicine. Expert panels operating under structured consensus protocols tend to distinguish between concerns that are ethically salient and those that are methodologically tractable within the evaluative frame being built. The exclusion of Domains F and G should therefore not be read as a denial of their normative importance, but as a realistic appraisal of the current boundary between what precision medicine HTA can coherently evaluate and what remains beyond its operational reach.

### 4.6. From Greece to Europe and Beyond: Implications and Transferability

The contribution of the study is methodological before it is substantive. It offers a transparent and resource-efficient consensus architecture that responds to the national mandate of the HTAR [10,19] and produces, as one application of that architecture, a domain-structured set of 15 ethical priorities calibrated to the Greek setting. The Greek context was not chosen arbitrarily. Greece is one of the more recent adopters of a structured HTA system in Europe and operates within institutional and resource conditions broadly representative of those facing other late-aligning Member States [20,21]. The findings are not generalisable to other EU systems in a strong sense; they are transferable insofar as those settings share comparable conditions, in line with Lincoln and Guba’s account of transferability in qualitative research [83].

The translational distance to operational use in Greece is shorter than is typical for academic consensus work. Two existing bodies hold competence through which the retained domains could be embedded into routine precision medicine HTA without new legislation or new institutional infrastructure [19,21]. EOPYY, as the primary reimbursement authority for medicines and health technologies, controls the submission requirements and assessment criteria through which a structured ethics component would enter operational practice. ODIPY, as the body responsible for quality assurance across the Greek health system, holds the standard-setting mandate that would give such a component normative standing. Three points of entry follow from this institutional surface: a structured ethics checklist appended to existing HTA submission requirements, a standing ethics advisory function within assessment committees, and a formal ethics annex to the national HTA report. Each is calibrated to a different level of institutional readiness and resource availability. An operationalised version of these three points of entry is provided in Supplementary Material S3, which maps each of the 15 retained statements to a specific assessment question, a documentation mechanism, and an outcome indicator—a ready-to-use instrument for HTA practitioners, assessment committees, and ethics advisory bodies.

The legal contour of the national mandate is sharper than the broad delegation language of the HTAR might suggest. Article 2(6) confines joint clinical assessments to clinical effectiveness and relative safety, while Article 13 places all non-clinical domains, including the ethical, organisational, social and legal, with national authorities [10]. The Regulation does not merely permit Member States to develop national ethics frameworks for precision medicine HTA; it requires them to, since the joint EU-level work will not produce outputs that fulfill this function. Several Member States already engaged in HTAR compliance report that capacity constraints, resource limitations and procedural gaps are the primary obstacles to meeting the regulation’s requirements at the national level [84]. The two-round Delphi process used here, with a multidisciplinary panel of 18 experts and CVR-based content validation, is calibrated to those constraints. It does not require the institutional infrastructure or funding of large multinational consensus processes such as those behind STANDING Together or FUTURE-AI [70,71].

This bears on a wider question about transferability. Kaló et al. argue, in the context of Central and Eastern European HTA, that uncritical transfer of evidence and frameworks across jurisdictions can do more harm than good, because the social, institutional and regulatory conditions shaping HTA practice differ substantially across countries [85]. Daubner-Bendes et al. document that HTA systems in the same region face shared methodological challenges in adapting assessment frameworks to their specific contexts, with mutual learning across newer and more established systems consistently identified as a pragmatic way forward [86]. Ethical frameworks are particularly sensitive to such variation, because their content is shaped by national institutional culture rather than by universal scientific facts. The methodological template offered here accommodates that sensitivity by design: it is the methodology, not the substantive set of statements, that is intended for adoption elsewhere. The transferable contribution of this study is methodological before it is substantive. The two-round Delphi architecture is calibrated to the resource and institutional constraints of late-aligning HTA systems and does not require the infrastructure of large multinational consensus processes. The two excluded domains were not excluded because the panel regarded them as unimportant; they were excluded because the methodological infrastructure for translating those values into assessable practice does not yet exist. The process required no travel, dedicated facilities, or external funding. Measured against the alternative—no ethics framework, or one transplanted from a different institutional context—the architecture is cost-necessary: the minimum viable methodological investment for meeting the Article 13 mandate with epistemic integrity rather than procedural compliance.

The relevance of the contribution extends beyond the EU. A 2025 WHO survey of HTA arrangements covering 104 countries found that approximately half (52%) have a legislative requirement to consider HTA results in coverage decisions [87]; across most of those countries, the ethical infrastructure for precision medicine HTA does not yet exist in any structured form. INAHTA, representing 53 member agencies across 34 countries and serving over 1 billion people [88], has no documented empirically grounded ethical framework specifically designed for precision medicine assessment. The Asia–Pacific region is engaging the same gap: the HTAsiaLink 2025 conference in Singapore dedicated a plenary session to the role of HTA in shaping AI-empowered personalized medicine, with equity and ethical implications identified as the central methodological challenge [89]. The Delphi architecture used here is therefore a candidate for application well beyond the setting in which it was generated.

Read against the broader project of integrating empirical analysis and normative inquiry in HTA articulated by VALIDATE [44,45] and by the HTAi Interest Group on Ethics in HTA, the present study makes a specific contribution. Van der Wilt et al. argue that methodological choices in HTA reflect normative commitments and that the relevance of evidence cannot be assessed independently of the values that guide its selection [45]. The Delphi framework reported here translates that recognition into a consensus-based set of ethical criteria for precision medicine assessment that can be applied in routine HTA work. The contribution is not the addition of ethics as a discrete domain alongside others, but the empirical specification of how ethical considerations can shape the conduct of HTA in a defined national context.

### 4.7. Limitations

This is a national proof-of-concept study. The expert panel was drawn exclusively from Greek academic, clinical and policy institutions, in line with the HTAR’s delegation of the ethical dimensions of HTA to national authorities. Generalisability beyond the Greek institutional context is not claimed. The findings should be read through transferability rather than statistical generalisability. As Lincoln and Guba argue, findings from a bounded context are not presumed to apply universally; they are offered as contextually described patterns, with judgement about applicability to other settings resting with the reader who knows the target context [83]. The priorities reported here are shaped by Greek professional culture, the institutional legacy of HTA development in the country, and its specific relationship with data governance and patient autonomy norms [20]. Cross-national replications are a natural extension of the work, not a precondition for its validity at the national level.

The relative weighting of the 15 priorities is sensitive to panel composition. A different balance of professions, higher patient representation, or experts drawn from other Member States could produce different orderings, and possibly different statements at the candidate-generation stage. This is a question for the multinational replication phase rather than a flaw of the national proof-of-concept reported here.

An 18-member panel sits within the range recommended for focused Delphi studies in health policy and HTA research [23,24,25,37,38,39], and the CVR threshold of 0.42 is statistically meaningful for N = 18 under Lawshe’s original derivation [27]. Panel size nonetheless constrains the disciplinary and institutional diversity that a larger or multinational panel could provide. Trustworthiness in qualitative and consensus research, as articulated by Nowell et al., rests on credibility, dependability, confirmability and transferability rather than on sample-size-based inference [40]. The present study addresses these criteria through methodological transparency, ACCORD-compliant reporting, and full documentation of the literature-informed approach to candidate statement generation in Supplementary Material S2.

Patient representation on the panel was limited to a single advocate (5.6%). The structural reasons for this pattern and its implications for the consensus on Domain B are discussed in Section 4.2. Future iterations of this methodology should incorporate co-creation with patients and the public from the item generation stage onward, for example through preliminary focus groups or co-design workshops, rather than limiting involvement to the validation phase. This would strengthen the ecological validity and democratic legitimacy of the resulting framework [30,52,53]. This recognition shapes the design priorities for the next phase of this research program: dedicated patient and public involvement (PPI) protocols developed in partnership with established patient organisations, applied during the item generation stage of any cross-national replication, with separate analytic treatment of patient-derived priorities to enable explicit comparison with professional consensus.

Open-ended responses collected in Round 1 were reviewed thematically to support the interpretation of domain-level findings. This qualitative analysis was used in a supplementary rather than primary capacity. Future iterations of the methodology would benefit from more structured integration of qualitative data, potentially using validated coding frameworks to enrich consensus interpretation. A further limitation concerns the three-point necessity scale. The strict CVR filter of Round 1 ensures that only statements with strong prior endorsement advance to Round 2; those statements are structurally predisposed to receive near-unanimous endorsement in a confirmatory round. The resulting ceiling—median 3.0, IQR 0.00 across all 15 retained statements—is an artefact of the design’s internal logic rather than a measurement failure. The framework identifies what Greek HTA experts regard as necessary, not which of those necessities they regard as more important. That question requires a subsequent study employing a five-point scale, pairwise comparison, or multi-criteria decision analysis weighting.

A final caveat concerns the provenance of the candidate statements pool. The 32 items were generated by the research team through the literature-informed approach documented in Section 2.2 and Supplementary Material S2 and cross-referenced against the EUnetHTA Core Model^®^ ethics domain [9] and the IC2PerMed governance roadmap [5]. This dual anchoring provides external warrant but does not eliminate the possibility that the candidate pool reflects the theoretical orientations of the investigators. A genuinely inductive item generation phase would not have imposed those boundaries. Future replications would benefit from preceding qualitative stages, including focus groups or interviews with HTA practitioners, patient representatives and policymakers, in which candidate statements are drawn from lived institutional experience before formal expert validation [23,24]. Such grounding would strengthen the ecological validity of the resulting framework for the national HTA settings in which it is intended to operate.

## 5. Conclusions

This study identified and prioritised 15 ethical considerations for health technology assessment in precision medicine. Consensus emerged across five thematic domains: fundamental ethical principles; transparency, stakeholder participation, and institutional accountability; equity and access; digital health and artificial intelligence; and pandemic preparedness and system resilience. The exclusion of environmental sustainability and social acceptability domains at content validation reflects the current methodological boundary of HTA-applicable ethical evaluation rather than a denial of their normative importance.

The study addresses the ethical mandate of the HTAR on two levels: a resource-efficient Delphi architecture suited to the capacity constraints of national HTA systems, and a substantive set of 15 priorities as one national instance of how that architecture produces operational guidance. The framework enters practice through three steps: a structured ethics checklist (Supplementary Material S3) appended to submission dossiers, ethics responses reviewed alongside clinical and economic analysis, and a dedicated ethics section in the final HTA report. None of these steps requires new legislation or additional resources. Cross-national replication, stronger patient and public involvement from the item generation stage, and further methodological development for currently immature domains (environmental sustainability and social acceptability) represent the natural next steps for this research program. As cross-national replications of this methodology accumulate, the resulting evidence base could inform future European-level coordination on the ethical dimensions of HTA, complementing rather than replacing the joint clinical assessment work coordinated under the HTAR [10].

## Figures and Tables

**Table 1:** Panel composition by professional role, field of expertise, institution type, and HTA/policy involvement (N = 18, anonymous presentation).

No.	Professional Role	Field of Expertise	Institution Type	HTA/Policy
1	Academic/Hospital Administrator	HTA/Health Management	University/Hospital	Yes
2	Academic (LabHTA)	HTA	University	Yes
3	Academic/Biomedical Engineer	Biomedical Technology/AI	Technical University	Yes
4	Academic (EDIP)	Public Health Policy	University	Yes
5	Professor/Agency President	Nursing/Quality Assurance	University/National body	Yes
6	Academic	Nursing/Health Services	University	Yes
7	Academic	Health Services Research/HTA	University	Yes
8	Researcher/CEO	Biomedical Technology	Research Institute	Yes
9	Professor	Law/Bioethics/Public Health	University	Yes
10	Associate Professor	Health Services/Health Economics	University	Yes
11	Professor	Health Policy	University	Yes
12	Professor	Health Policy	University	Yes
13	Professor	Philosophy/Ethics	University	Partial
14	Academic	Nursing/Health Economics	University	Yes
15	Senior Officer	Pharmaceutical Policy	National body (EOPYY)	Yes
16	Researcher	Pharmacoeconomics/HTA	University	Yes
17	Lawyer/Patient Advocate	Law/Patient Representation	Patient association	Yes
18	Academic	Public Health/Health Policy	University	Yes

Note: Numbering is arbitrary and does not reflect order of recruitment. HTA = Health Technology Assessment. Yes = documented involvement in HTA or health policy activities. Partial = involvement in related but not directly HTA-specific activities.

**Table 2:** Round 2 consensus results for the 15 retained ethical statements (N = 18 experts).

Code	Statement	Median	IQR	% Agreement
**Domain A: Fundamental Ethical Principles**				
**A1**	Justice and equality should constitute fundamental principles in every HTA procedure.	3.0	0.00	100.0%
**A2**	Respect for human dignity should prevail over purely economic parameters.	3.0	0.00	100.0%
**A4**	Non-maleficence and beneficence should be incorporated as criteria in HTA.	3.0	0.00	100.0%
**Domain B: Transparency, Stakeholder Participation, and Institutional Accountability**				
**B1**	Patient and citizen participation should be mandatory at all stages of HTA.	3.0	0.00	94.4%
**B2**	HTA procedures should be transparent and all criteria/data should be publicly disclosed.	3.0	0.00	100.0%
**B3**	A statutory mechanism for objections and review of HTA decisions should exist.	3.0	0.00	100.0%
**B5**	Professional training in HTA ethics should become a statutory requirement.	3.0	0.00	100.0%
**Domain C: Equity and Access**				
**C1**	Equal access to health services should constitute a criterion in assessments.	3.0	0.00	100.0%
**C4**	Health quality indicators should be published disaggregated by social group.	3.0	0.00	100.0%
**Domain D: Digital Health and Artificial Intelligence**				
**D1**	Digital health technologies should also be evaluated on the basis of ethical criteria.	3.0	0.00	100.0%
**D2**	AI technologies should be systematically checked for bias both before approval and during clinical use.	3.0	0.00	100.0%
**D3**	The explainability of AI systems should be a prerequisite for HTA.	3.0	0.00	100.0%
**D4**	There should always be human oversight in decisions taken with AI support.	3.0	0.00	100.0%
**D5**	The degree of digital divide should be assessed.	3.0	0.00	100.0%
**Domain E: Pandemic Preparedness and System Resilience**				
**E2**	System readiness for supply chain disruptions or pandemics should be assessed within HTA.	3.0	0.00	100.0%

Note: Consensus criteria: median ≥ 2.0 and ≥80% agreement in the top two response categories. IQR = interquartile range. All 15 retained statements achieved consensus in Round 2. The three-point scale used: 1 = Not necessary; 2 = Useful but not necessary; 3 = Necessary.

**Table 3:** Comparison of existing ethical frameworks for HTA with the present study.

Feature	EUnetHTA Core Model^®^ (ETH Domain)	ICER Special Ethical Priorities (2023)	FUTURE-AI/STANDING Together	This Study
Primary focus	Six ethical categories (benefit–harm balance, autonomy, respect for persons, justice and equity, legislation, ethical consequences of HTA)	Supplementary ethical criteria within QALY framework	AI bias monitoring, transparency, explainability, and human oversight	Domain-structured substantive ethical priorities for precision medicine HTA
Methodological basis	Expert consensus and assessment-element framework	Institutional review and stakeholder input	International multi-stakeholder consensus	Modified two-round Delphi with CVR-based content validation
Precision medicine specificity	Generic across technology types	Generic across technology types	AI-specific, not precision-medicine-specific	Calibrated to precision medicine
HTAR alignment	Foundational reference, but does not address national mandate under HTAR Article 13	Outside EU regulatory framework	Sectoral, not aligned with HTAR domains	Operationally aligned with HTAR Article 13 ethical-domain responsibilities
Output	Conceptual map and structured question set; not operationalised for precision medicine	Supplementary criteria list	Sector-specific guidelines	15 prioritised ethical statements across 5 domains

Note: EUnetHTA = European Network for Health Technology Assessment; ETH = ethical analysis domain of the EUnetHTA Core Model; ICER = Institute for Clinical and Economic Review; FUTURE-AI = international consensus guideline for trustworthy and deployable AI in healthcare; STANDING Together = standards for data diversity, inclusivity and generalizability; HTAR = EU Regulation 2021/2282 on Health Technology Assessment; CVR = Content Validity Ratio.

## Data Availability

The anonymized data supporting the findings of this study are available from the corresponding author upon reasonable request. Individual-level responses cannot be made publicly available due to the commitments of participant anonymity established during the informed consent process.

## Supplementary Materials

The following supporting information can be downloaded at: https://www.mdpi.com/article/10.3390/jpm16060308/s1, Table S1: Content validity results for all 32 candidate ethical statements (Round 1, N = 18), reporting the number of expert ratings per response category, the content validity ratio (CVR), the percentage agreement, and the retention outcome for each statement; Supplementary Material S2: Systematic review of methodological guidance on the integration of ethics into HTA, documenting the search strategy, screening process, PRISMA 2020 flow diagram, the included studies [1,46,90,91,92,93,94,95,96,97,98,99,100,101,102,103,104,105,106,107,108,109], and the thematic mapping to the candidate item pool; Supplementary Material S3: HTA Ethics Checklist for Precision Medicine—an operational companion to the Delphi-derived framework that decomposes the 15 retained ethical priorities into specific assessment items.

## Abbreviations

The following abbreviations are used in this manuscript:

HTA	Health Technology Assessment
AI	Artificial Intelligence
NICE	National Institute for Clinical Excellence
HAS	Haute Autorité de santé
IQWiG	Institute for Quality and Efficiency in Health Care
HTAR	Health Technology Assessment Regulation
EU	European Union
ICER	Institute for Clinical and Economic Review
IC2PerMed	International Consortium for Personalized Medicine
GDPR	General Data Protection Regulation
CVR	Content Validity Ratio
QALY	Quality-Adjusted Life Year
OECD	Organization for Economic Co-operation and Development
ACCORD	Accurate Consensus Reporting Document
