# Supplementary material for: Ethical Considerations in Health Technology Assessment for Precision Medicine: A Delphi Study in a Greek Setting
Source: J Pers Med. 2026 Jun 5;16(6):308. doi: 10.3390/jpm16060308 (PMC13301307; doi:10.3390/jpm16060308)
Supplement: Supplementary file 1 [file jpm-16-00308-s001.zip › ACCORD_Checklist.docx]

**ACCORD Checklist**

ACcurate COnsensus Reporting Document (Gattrell et al., PLoS Med 2024)

Manuscript: Ethics Considerations in Health Technology Assessment for Personalized Medicine: A Delphi Study

| **Item** | **Checklist Item** | **Location in Manuscript** | **Reported** |
| --- | --- | --- | --- |
| **TITLE** | | | |
| **T1** | Indicate in the title that consensus methods were used | *Title* | **Yes** |
| **INTRODUCTION** | | | |
| **I1** | State the objective of the consensus exercise | *Section 1 (para 4)* | **Yes** |
| **I2** | Describe why a consensus approach was chosen | *Section 2.1* | **Yes** |
| **I3** | Identify the target users of the consensus output | *Section 1 (para 4), 4.6* | **Yes** |
| **METHODS** | | | |
| **M1** | Identify the consensus method used (e.g., Delphi, nominal group technique) | *Section 2.1* | **Yes** |
| **M2** | Describe the process used to select and invite participants | *Section 2.3* | **Yes** |
| **M3** | State the inclusion and exclusion criteria for participants | *Section 2.3* | **Yes** |
| **M4** | Describe how many participants were targeted and why | *Section 2.3* | **Yes** |
| **M5** | Describe the professional background/expertise of participants | *Section 2.3* | **Yes** |
| **M6** | State the number of participants who were approached, agreed to participate, and completed each round | *Section 2.3* | **Yes** |
| **M7** | Describe the information sources or methods used to generate the initial list of items | *Section 2.2* | **Yes** |
| **M8** | Describe how the initial list of items was developed | *Section 2.2* | **Yes** |
| **M9** | Describe how participants were briefed about the consensus exercise | *Section 2.5* | **Yes** |
| **M10** | State the number of rounds conducted | *Section 2.1* | **Yes** |
| **M11** | Describe the mode of communication used in each round (online, in-person, etc.) | *Section 2.4* | **Yes** |
| **M12** | Describe what participants were asked to do in each round | *Section 2.1, 2.4* | **Yes** |
| **M13** | Describe the rating/voting scale used | *Section 2.1* | **Yes** |
| **M14** | State the pre-specified consensus definition and threshold | *Section 2.1* | **Yes** |
| **M15** | Describe what feedback was provided to participants between rounds | *Section 2.4* | **Yes** |
| **M16** | Describe how the results of each round were analysed | *Section 2.4* | **Yes** |
| **M17** | Describe how items were retained, modified, or excluded between rounds | *Section 2.1* | **Yes** |
| **M18** | State whether anonymity of participants was maintained and how | *Section 2.4, 2.5* | **Yes** |
| **M19** | Describe any pilot testing of the questionnaire | *Section 2.2 (internal review as pragmatic pilot substitute)* | **Partial** |
| **M20** | State any changes made to the protocol after the study began | *N/A — no protocol changes* | N/A |
| **M21** | State the ethical approval obtained and participant consent procedures | *Section 2.5* | **Yes** |
| **RESULTS** | | | |
| **R1** | Report response rates and completion for each round | *Section 2.3, 3. Results* | **Yes** |
| **R2** | Describe participant characteristics | *Section 2.3* | **Yes** |
| **R3** | Report the results of each consensus round | *Section 3. Results* | **Yes** |
| **R4** | Report the final consensus outcome | *Section 3. Results, Table 1* | **Yes** |
| **R5** | Report any deviations from the pre-specified protocol | *N/A — no deviations* | N/A |
| **DISCUSSION** | | | |
| **D1** | Discuss the strengths and limitations of the consensus exercise | *Section 4.7* | **Yes** |
| **D2** | Discuss the implications of the consensus output and recommendations for future work | *Section 4.6, 5. Conclusions* | **Yes** |
| **OTHER** | | | |
| **O1** | List any endorsing organisations involved and their role | *N/A* | N/A |
| **O2** | State if and where the study was registered | *N/A — not required for Delphi studies* | N/A |
| **O3** | State any funding received and the role of the funder | *Back matter — This research received no external funding* | **Yes** |

Note: Green = Reported; Grey = Not applicable.
