# Supplementary material for: Ethical Considerations in Health Technology Assessment for Precision Medicine: A Delphi Study in a Greek Setting
Source: J Pers Med. 2026 Jun 5;16(6):308. doi: 10.3390/jpm16060308 (PMC13301307; doi:10.3390/jpm16060308)
Supplement: Supplementary file 1 [file jpm-16-00308-s001.zip › Supplementary_Table_S1.docx]

**Supplementary Table S1.** Content Validity Results for All 32 Candidate Ethical Statements (Round 1, N = 18)

***Note:*** *Ne = number of experts rating the statement as "Necessary"; Nu = "Useful but not necessary"; Nn = "Not necessary". CVR = Content Validity Ratio (Lawshe, 1975): CVR = (Ne − N/2) / (N/2). Retention criteria: CVR ≥ 0.42 AND ≥ 80% agreement in top two response categories. Retained statements are highlighted in green; excluded statements in yellow.*

| **Code** | **Statement** | **Ne** | **Nu** | **Nn** | **CVR** | **% Agreement** | **Outcome** |
| --- | --- | --- | --- | --- | --- | --- | --- |
| **Domain A: Fundamental Ethical Principles** | | | | | | | |
| **A1** | Justice and equality should constitute fundamental principles in every HTA procedure. | 18 | 0 | 0 | 1.000 | 100.0% | **Retained** |
| **A2** | Respect for human dignity should prevail over purely economic parameters. | 13 | 5 | 0 | 0.444 | 100.0% | **Retained** |
| A3 | Patient autonomy should be taken into account in assessments. | 12 | 6 | 0 | 0.333 | 100.0% | Excluded |
| **A4** | Non-maleficence and beneficence should be incorporated as criteria in HTA. | 18 | 0 | 0 | 1.000 | 100.0% | **Retained** |
| **Domain B: Transparency, Stakeholder Participation & Institutional Accountability** | | | | | | | |
| **B1** | Patient and citizen participation should be mandatory at all stages of HTA. | 13 | 5 | 0 | 0.444 | 100.0% | **Retained** |
| **B2** | HTA procedures should be transparent and all criteria/data should be publicly disclosed. | 14 | 4 | 0 | 0.556 | 100.0% | **Retained** |
| **B3** | A statutory mechanism for objections and review of HTA decisions should exist. | 14 | 4 | 0 | 0.556 | 100.0% | **Retained** |
| B4 | Conflicts of interest of those participating in HTA should be mandatorily disclosed. | 12 | 6 | 0 | 0.333 | 100.0% | Excluded |
| **B5** | Professional training in HTA ethics should become a statutory requirement. | 13 | 5 | 0 | 0.444 | 100.0% | **Retained** |
| **Domain C: Equity and Access** | | | | | | | |
| **C1** | Equal access to health services should constitute a criterion in assessments. | 17 | 1 | 0 | 0.889 | 100.0% | **Retained** |
| C2 | Assessments should take into account vulnerable groups (migrants, persons with disabilities, low income). | 12 | 6 | 0 | 0.333 | 100.0% | Excluded |
| C3 | Equity indicators (e.g., language, socioeconomic status, geographic remoteness) should be systematically collected. | 11 | 6 | 1 | 0.222 | 94.4% | Excluded |
| **C4** | Health quality indicators should be published disaggregated by social group. | 13 | 3 | 2 | 0.444 | 88.9% | **Retained** |
| C5 | Priority should be given to younger patients over older ones in cases of limited resources. | 2 | 7 | 9 | -0.778 | 50.0% | Excluded |
| C6 | Therapies with excessively high costs should be rejected even if they are the only option for few patients (e.g., orphan drugs). | 1 | 6 | 11 | -0.889 | 38.9% | Excluded |
| C7 | HTA decisions should be based primarily on utilitarian approaches. | 1 | 14 | 3 | -0.889 | 83.3% | Excluded |
| C8 | A hybrid framework (combination of utilitarianism and ethics of care) is more appropriate for HTA procedures. | 10 | 6 | 2 | 0.111 | 88.9% | Excluded |
| C9 | Personalized care and patient autonomy should take priority over utility. | 5 | 11 | 2 | -0.444 | 88.9% | Excluded |
| **Domain D: Digital Health and Artificial Intelligence** | | | | | | | |
| **D1** | Digital health technologies should also be evaluated on the basis of ethical criteria. | 15 | 3 | 0 | 0.667 | 100.0% | **Retained** |
| **D2** | AI technologies should be systematically checked for bias both before approval and during clinical use. | 15 | 3 | 0 | 0.667 | 100.0% | **Retained** |
| **D3** | The explainability of AI systems should be a prerequisite for HTA. | 13 | 5 | 0 | 0.444 | 100.0% | **Retained** |
| **D4** | There should always be human oversight in decisions taken with AI support. | 14 | 4 | 0 | 0.556 | 100.0% | **Retained** |
| **D5** | The degree of digital divide (e.g., access to digital infrastructure) should be assessed. | 13 | 5 | 0 | 0.444 | 100.0% | **Retained** |
| D6 | It is acceptable to adopt AI that operates as a "black box" if it is clinically effective. | 3 | 9 | 6 | -0.667 | 66.7% | Excluded |
| **Domain E: Pandemic Preparedness and System Resilience** | | | | | | | |
| E1 | HTA should include resilience criteria for health system preparedness in public health crises. | 11 | 6 | 1 | 0.222 | 94.4% | Excluded |
| **E2** | System readiness for supply chain disruptions or pandemics should be assessed within HTA. | 14 | 4 | 0 | 0.556 | 100.0% | **Retained** |
| **Domain F: Environmental Sustainability** | | | | | | | |
| F1 | The environmental footprint (carbon footprint, waste) should constitute an official HTA criterion. | 7 | 7 | 4 | -0.222 | 77.8% | Excluded |
| F2 | Technologies with a high environmental footprint should be rejected even if clinically effective. | 5 | 8 | 5 | -0.444 | 72.2% | Excluded |
| F3 | Healthcare procurement procedures should take into account green sustainability. | 11 | 6 | 1 | 0.222 | 94.4% | Excluded |
| **Domain G: Social Acceptability and Public Trust** | | | | | | | |
| G1 | Social acceptability of a new technology by society should be taken into account. | 7 | 9 | 2 | -0.222 | 88.9% | Excluded |
| G2 | Public trust in the health system should be considered a quality indicator of HTA. | 11 | 4 | 3 | 0.222 | 83.3% | Excluded |
| G3 | Social impacts (e.g., isolation of elderly due to robotic care) should be evaluated on equal terms with clinical outcomes. | 12 | 5 | 1 | 0.333 | 94.4% | Excluded |
