# Supplementary material for: Ethical Considerations in Health Technology Assessment for Precision Medicine: A Delphi Study in a Greek Setting
Source: J Pers Med. 2026 Jun 5;16(6):308. doi: 10.3390/jpm16060308 (PMC13301307; doi:10.3390/jpm16060308)
Supplement: Supplementary file 1 [file jpm-16-00308-s001.zip › ACCORD_Checklist.pdf]

ACCORD Checklist

ACcurate COnsensus Reporting Document (Gattrell et al., PLoS Med 2024)

Manuscript: Ethics Considerations in Health Technology Assessment for Personalized Medicine: A Delphi Study

Item	Checklist Item	Location in Manuscript	Reported
**TITLE**			
**T1**	Indicate in the title that consensus methods were used	*Title*	Yes
**INTRODUCTION**			
**I1**	State the objective of the consensus exercise	*Section 1 (para 4)*	Yes
**I2**	Describe why a consensus approach was chosen	*Section 2.1*	Yes
**I3**	Identify the target users of the consensus output	*Section 1 (para 4), 4.6*	Yes
**METHODS**			
**M1**	Identify the consensus method used (e.g., Delphi, nominal group technique)	*Section 2.1*	Yes
**M2**	Describe the process used to select and invite participants	*Section 2.3*	Yes
**M3**	State the inclusion and exclusion criteria for participants	*Section 2.3*	Yes
**M4**	Describe how many participants were targeted and why	*Section 2.3*	Yes
**M5**	Describe the professional background/expertise of participants	*Section 2.3*	Yes
**M6**	State the number of participants who were approached, agreed to participate, and completed each round	*Section 2.3*	Yes
**M7**	Describe the information sources or methods used to generate the initial list of items	*Section 2.2*	Yes
**M8**	Describe how the initial list of items was developed	*Section 2.2*	Yes
**M9**	Describe how participants were briefed about the consensus exercise	*Section 2.5*	Yes
**M10**	State the number of rounds conducted	*Section 2.1*	Yes
**M11**	Describe the mode of communication used in each round (online, in-person, etc.)	*Section 2.4*	Yes
**M12**	Describe what participants were asked to do in each round	*Section 2.1, 2.4*	Yes
**M13**	Describe the rating/voting scale used	*Section 2.1*	Yes
**M14**	State the pre-specified consensus definition and threshold	*Section 2.1*	Yes
**M15**	Describe what feedback was provided to participants between rounds	*Section 2.4*	Yes
**M16**	Describe how the results of each round were analysed	*Section 2.4*	Yes
**M17**	Describe how items were retained, modified, or excluded between rounds	*Section 2.1*	Yes
**M18**	State whether anonymity of participants was maintained and how	*Section 2.4, 2.5*	Yes
**M19**	Describe any pilot testing of the questionnaire	*Section 2.2 (internal review as pragmatic pilot substitute)*	~~Yes~~Partial
**M20**	State any changes made to the protocol after the study began	*N/A — no protocol changes*	N/A
**M21**	State the ethical approval obtained and participant consent procedures	*Section 2.5*	Yes

**RESULTS**			
**R1**	Report response rates and completion for each round	*Section 2.3, 3. Results*	**Yes**
**R2**	Describe participant characteristics	*Section 2.3*	**Yes**
**R3**	Report the results of each consensus round	*Section 3. Results*	**Yes**
**R4**	Report the final consensus outcome	*Section 3. Results, Table 1*	**Yes**
**R5**	Report any deviations from the pre-specified protocol	*N/A — no deviations*	N/A
**DISCUSSION**			
**D1**	Discuss the strengths and limitations of the consensus exercise	*Section 4.7*	**Yes**
**D2**	Discuss the implications of the consensus output and recommendations for future work	*Section 4.6, 5. Conclusions*	**Yes**
**OTHER**			
**O1**	List any endorsing organisations involved and their role	*N/A*	N/A
**O2**	State if and where the study was registered	*N/A — not required for Delphi studies*	N/A
**O3**	State any funding received and the role of the funder	*Back matter — This research received no external funding*	**Yes**

Note: Green = Reported; Grey = Not applicable.
