# Supplementary material for: Ethical Considerations in Health Technology Assessment for Precision Medicine: A Delphi Study in a Greek Setting
Source: J Pers Med. 2026 Jun 5;16(6):308. doi: 10.3390/jpm16060308 (PMC13301307; doi:10.3390/jpm16060308)
Supplement: Supplementary file 1 [file jpm-16-00308-s001.zip › Supplementary_S2.pdf]

Supplementary Material S2

Systematic Review of Methodological Guidance on the Integration of Ethics into HTA: Documentation of Search Strategy and Included Studies

S2.1. Overview

The candidate ethical statements submitted to the Delphi panel in the present study were generated through a structured systematic review of methodological guidance on the integration of ethics into Health Technology Assessment (HTA), conducted by the research team in accordance with the PRISMA 2020 guidelines [S1].

The systematic review aimed to identify and analyse current methodological approaches to ethical evaluation within HTA frameworks, with particular attention to emerging technologies (artificial intelligence, robotics, environmentally sustainable innovations), patient and stakeholder participation, and equity considerations. Twenty-one studies meeting the inclusion criteria were retained for analysis, and the recurring ethical themes identified across these studies informed the construction of the 32 candidate statements that were subsequently evaluated by the multidisciplinary expert panel.

This Supplementary Material documents the search strategy, screening process, PRISMA flow, included studies, and thematic mapping that connects the systematic review to the candidate item pool of the main study.

S2.2. Search Strategy

Databases

Three primary biomedical and multidisciplinary databases were searched:

- PubMed (MEDLINE)
- Embase
- Google Scholar

Time period

January 2019 to December 2024. The five-year window was selected to capture recent methodological developments in HTA ethics, including those responding to the rapid expansion of artificial intelligence in healthcare.

Search execution

Searches were conducted between September and October 2024.

Search terms

The full search strategy combined Medical Subject Headings (MeSH) and free-text terms organised around three thematic blocks: Health Technology Assessment, Ethical Considerations, and Methodological Guidance. Table S2.1 reports the complete MeSH-based search strategy.

Table S2.1. Medical Subject Headings (MeSH) Search Strategy.

Concept block	Search terms
**Health Technology Assessment**	"Technology assessment, biomedical" [MeSH] OR ("technology" [all fields] AND "assessment" [all fields] AND "biomedical" [all fields]) OR "biomedical technology assessment" [all fields] OR ("health" [all fields] AND "technology" [all fields] AND "assessment" [all fields]) OR "health technology assessment" [all fields]
**Medical Device**	"Equipment and supplies" [MeSH] OR ("equipment" [all fields] AND "supplies" [all fields]) OR "equipment and supplies" [all fields] OR ("medical" [all fields] AND "device" [all fields]) OR "medical device" [all fields]
**Artificial Intelligence**	"Artificial intelligence" [MeSH] OR ("artificial" [all fields] AND "intelligence" [all fields]) OR "artificial intelligence" [all fields]
**Ethical Considerations**	"Ethics" [MeSH] OR "bioethics" [MeSH] OR "ethical analysis" [all fields] OR "ethical considerations" [all fields] OR "ethical aspects" [all fields]
**Methodological Guidance**	"Practice Guidelines as Topic" [MeSH] OR "methodology" [all fields] OR "framework" [all fields] OR "guidance" [all fields] OR "methodological" [all fields]

S2.3. Inclusion and Exclusion Criteria

Inclusion criteria

• Peer-reviewed publication

• Published in English

• Published between January 2019 and December 2024

• Methodological focus on ethical aspects of health technology assessment

• Original empirical study, methodological framework, or applied HTA report explicitly addressing ethical aspects

Exclusion criteria

• Sources outside the focus of the review (e.g., not addressing methodological aspects of ethics in HTA)

• Non-scientific or non-regulatory publications

• Editorials, letters to the editor, and conference abstracts

• Articles without original data or analysis on ethical measures in health technology

S2.4. PRISMA 2020 Flow Diagram

The flow of records through the screening process is summarised below.

IDENTIFICATION	
**Records identified from databases**<br>PubMed, Embase, Google Scholar (n = 1,240)	**Records removed before screening:**<br>• Duplicates (n = 560)<br>• Marked ineligible by automation tools (n = 20)<br>• Removed for other reasons (n = 10)
**SCREENING**	
**Records after initial filters**<br>(n = 650)	Records excluded by title and abstract (n = 555)
**Full-text articles assessed for eligibility**<br>(n = 95)	**Full-text articles excluded (n = 74):**<br>• Lack of ethical concern (n = 30)<br>• Poor methodological reporting (n = 25)<br>• Duplicate systematic reviews (n = 19)
**INCLUDED**	
**Studies included in the systematic review**<br>**(n = 21)**	

Figure S2.1. *PRISMA 2020 flow diagram for the systematic review of methodological guidance on the integration of ethics into HTA. The diagram reports identification, screening, eligibility, and inclusion phases following PRISMA 2020 reporting standards [S1].*

S2.5. List of Included Studies (n = 21)

The 21 studies retained for analysis are listed below. They were selected for their methodological focus on ethical evaluation in HTA and for their representation of recent developments in the field.

S2. Vreman, R.A.; Mantel-Teeuwisse, A.K.; Hövels, A.M.; Leufkens, H.G.M.; Goettsch, W.G. Differences in health technology assessment recommendations among European jurisdictions: the role of practice variations. Value Health 2020, 23(1), 10–16. https://doi.org/10.1016/j.jval.2019.07.017.

S3. O'Rourke, B.; Oortwijn, W.; Schuller, T. The new definition of health technology assessment: A milestone in international collaboration. Int. J. Technol. Assess. Health Care 2020, 36, 187–190. https://doi.org/10.1017/S0266462320000215.

S4. Pascal, C.; Mathy, C.; Bongiovanni, I.; Konishi, M. Integrating organizational impacts into health technology assessment (HTA): an analysis of the content and use of existing evaluation frameworks. Int. J. Technol. Assess. Health Care 2022, 38(1), e80. https://doi.org/10.1017/S0266462322003221.

S5. Bertram, M.; Dhaene, G.; Tan-Torres Edejer, T. (Eds.) Institutionalizing Health Technology Assessment Mechanisms: A How-to Guide; World Health Organization: Geneva, Switzerland, 2021; ISBN 978-92-4-002066-5. Available online: https://www.who.int/publications/i/item/9789240020665 (accessed on 30 April 2026).

S6. Kamaruzaman, H.F.; Grieve, E.; Wu, O. Disinvestment in healthcare: a scoping review of systematic reviews. Int. J. Technol. Assess. Health Care 2022, 38(1), e69. https://doi.org/10.1017/S0266462322000514.

S7. Richardson, J.; Schlander, M. Health technology assessment (HTA) and economic evaluation: efficiency or fairness first. J. Mark. Access Health Policy 2018, 7, 1557981. https://doi.org/10.1080/20016689.2018.1557981.

S8. Ekmekci, P.E.; Güner, M.D. Evaluation of ethical analyses in seven reports from the European Network for Health Technology Assessment. Int. J. Technol. Assess. Health Care 2019, 35(4), 273–279. https://doi.org/10.1017/S0266462319000485.

S9. Liyanage, H.; Liaw, S.T.; Jonnagaddala, J.; Schreiber, R.; Kuziemsky, C.; Terry, A.L.; de Lusignan, S. Artificial intelligence in primary health care: perceptions, issues, and challenges. Yearb. Med. Inform. 2019, 28(1), 41–46. https://doi.org/10.1055/s-0039-1677901.

S10. Polus, S.; Mathes, T.; Klingler, C.; Messer, M.; Gerhardus, A.; Stegbauer, C.; Willms, G.; Ehrenreich, H.; Marckmann, G.; Pieper, D. Health technology assessment of public health interventions published 2012 to 2016: an analysis of characteristics and comparison of methods. Int. J. Technol. Assess. Health Care 2019, 35(4), 280–290. https://doi.org/10.1017/S0266462319000515

S11. Bélisle-Pipon, J.C.; Couture, V.; Roy, M.C.; Ganache, I.; Goetghebeur, M.; Cohen, I.G. What makes artificial intelligence ex-ceptional in health technology assessment? Front. Artif. Intell. 2021, 4, 736697. https://doi.org/10.3389/frai.2021.736697.

S12. Otto, I.; Kahrass, H.; Mertz, M. “Same same but different”? On the questionable but crucial differentiation of ethical and social aspects in health technology assessment. Z. Evid. Fortbild. Qual. Gesundhwes. 2021, 164, 1–10. https://doi.org/10.1016/j.zefq.2021.05.007.

S13. Teerawattananon, Y.; Painter, C.; Dabak, S.; Ottersen, T.; Gopinathan, U.; Chola, L.; Chalkidou, K.; Culyer, A.J. Avoiding health technology assessment: a global survey of reasons for not using health technology assessment in decision making. Cost Eff. Resour. Alloc. 2021, 19(1), 62. https://doi.org/10.1186/s12962-021-00308-1.

S14. De Simone, P.; Ghinolfi, D. Hospital-based health technology assessment of machine perfusion systems for human liver trans-plantation. Transpl. Int. 2022, 35, 10405. https://doi.org/10.3389/ti.2022.10405.

S15. Farkowski, M.M.; Lach, K.; Pietrzyk, M.; Baryla-Zapala, E.; Gałązka-Sobotka, M.; Kowalska-Bobko, I.; Kępka, C.; Hryniewiecki, T. The Need to Implement Health Technology Assessment in Polish Hospitals—A Survey of 50 Hospital Managers. Int. J. En-viron. Res. Public Health 2022, 19, 8855. https://doi.org/10.3390/ijerph19148855.

S16. Guirado-Fuentes, C.; Abt-Sacks, A.; Trujillo-Martín, M.D.M.; García-Pérez, L.; Rodríguez-Rodríguez, L.; Carrion i Ribas, C.; Serrano-Aguilar, P. Main challenges of incorporating environmental impacts in the economic evaluation of health technology assessment: a scoping review. Int. J. Environ. Res. Public Health 2023, 20(6), 4949. https://doi.org/10.3390/ijerph20064949

S17. Holtorf, A.P.; Danyliv, A.; Krause, A.; Hanna, A.; Venable, Y.; Mattingly, T. J. 2nd; Huang, L.Y.; Pierre, M.; Silveira, Silva. A.; Walsh, D. Ethical and legal considerations in social media research for health technology assessment: conclusions from a scoping review. Int J Technol Assess Health Care. 2023 Oct 16;39(1):e62. doi: 10.1017/S0266462323000399. PMID: 37842838; PMCID: PMC11570170.

S18. Monleón, C.; Martin-Späth, H.; Crespo, C.; Dussart, C.; Toumi, M. Implicit factors influencing the HTA deliberative processes in 5 European countries: results from a mixed-methods research. Health Policy OPEN 2023, 5, 100109. https://doi.org/10.1016/j.hpopen.2023.100109.

S19. Torres-Castaño, A.; Abt-Sacks, A.; Toledo-Chávarri, A.; Suarez-Herrera, J.C.; Delgado-Rodríguez, J.; León-Salas, B.; Gonzá-lez-Hernández, Y.; Carmona-Rodríguez, M.; Serrano-Aguilar, P. Ethical, legal, organisational and social

issues of teleneurology: a scoping review. Int. J. Environ. Res. Public Health 2023, 20(4), 3694. https://doi.org/10.3390/ijerph20043694.

S20. Farah, L.; Borget, I.; Martelli, N.; Vallée, A. Suitability of the current health technology assessment of innovative artificial in-telligence-based medical devices: scoping literature review. J. Med. Internet Res. 2024, 26, e51514. https://doi.org/10.2196/51514.

S21. Lipska, I.; Di Bidino, R.; Niewada, M.; Nemeth, B.; Bochenek, T.; Kukla, M.; Więckowska, B.; Sobczak, A.; Iłowiecka, K.; Zemplenyi, A.; et al. Overcoming barriers in hospital-based health technology assessment (HB-HTA): international expert panel consensus. Healthcare (Basel) 2024, 12(9), 889. https://doi.org/10.3390/healthcare12090889.

S22. Palmier, C.; Rigaud, A.S.; Ogawa, T.; Wieching, R.; Dacunha, S.; Barbarossa, F.; Stara, V.; Bevilacqua, R.; Pino, M. Identification of ethical issues and practice recommendations regarding the use of robotic coaching solutions for older adults: narrative review. J. Med. Internet Res. 2024, 26, e48126. https://doi.org/10.2196/48126.

S2.6. Mapping from Systematic Review Findings to Candidate Statement Domains

The thematic content of the 21 included studies was mapped onto seven candidate ethical domains for the present Delphi study. Table S2.2 summarises the mapping between recurring themes identified in the systematic review and the seven candidate domains.

Table S2.2. Mapping of systematic review themes to candidate ethical domains.

Domain	Domain title	Recurring themes from systematic review
**A**	Fundamental ethical principles	Justice, autonomy, dignity, beneficence, non-maleficence, fairness in resource allocation
**B**	Transparency, stakeholder participation, and institutional accountability	Procedural transparency, deliberative legitimacy, institutional review processes, public accountability, conflict of interest
**C**	Equity and access	Disparities in access to health technologies, vulnerable populations, equitable distribution of benefits, contextual differences across health systems
**D**	Digital health and artificial intelligence	Algorithmic bias, AI explainability, data governance, human oversight in AI-supported decisions, privacy in digital health, the digital divide
**E**	Pandemic preparedness and system resilience	Health system resilience, ethical implications of supply chain disruption, distributive justice in crisis settings
**F**	Environmental sustainability	Ecological footprint of health technologies, life-cycle environmental assessment, sustainability in HTA
**G**	Social acceptability and public trust	Public engagement, social legitimacy of HTA decisions, trust in health technology governance

S2.7. Reporting Standard

The systematic review was conducted and reported in accordance with the PRISMA 2020 statement [S1].

S1. Page, M.J.; McKenzie, J.E.; Bossuyt, P.M.; Boutron, I.; Hoffmann, T.C.; Mulrow, C.D.; Shamseer, L.; Tetzlaff, J.M.; Akl, E.A.; Brennan, S.E.; et al. The PRISMA 2020 statement: an updated guideline for reporting systematic reviews. BMJ 2021, 372, n71. https://doi.org/10.1136/bmj.n71.
