# Supplementary material for: Ethical Considerations in Health Technology Assessment for Precision Medicine: A Delphi Study in a Greek Setting
Source: J Pers Med. 2026 Jun 5;16(6):308. doi: 10.3390/jpm16060308 (PMC13301307; doi:10.3390/jpm16060308)
Supplement: Supplementary file 1 [file jpm-16-00308-s001.zip › Supplementary_S3.pdf]

Supplementary Material S3

HTA Ethics Checklist for Precision Medicine: An Operational Companion to the Delphi-Derived Framework

This checklist operationalises the 15 ethical priorities identified through the modified two-round Delphi study reported in the main manuscript. Each priority is decomposed into one or more specific assessment items that an HTA practitioner should address when conducting the ethical evaluation of a precision medicine technology. The checklist is intended as a working aid, not a substitute for substantive ethical analysis.

Format. The format follows the conventions of established reporting checklists in biomedical research (CONSORT, STROBE, PRISMA, ACCORD). For each item, the assessor records: (i) whether the item was addressed in the assessment (Yes / Partial / No / Not applicable), (ii) the specific section or page of the assessment report where the item is documented, and (iii) any explanatory comments or evidence. Gaps and partial responses are themselves analytically informative and should be discussed in the final assessment report. The checklist is not a scoring instrument and does not produce an aggregate score.

Use within the HTA workflow. The checklist is intended for completion during the body of the assessment, with a final review at the recommendation stage. Items left unaddressed at the conclusion of the assessment should be reported transparently in the final HTA report, with reasoning.

Domain colour coding. Domain A and Domain B are highlighted in purple (foundational principles); Domain C and Domain E in teal (distributive concerns); Domain D in amber (technological frontier). The colour scheme matches Figure 2 of the main manuscript.

Domain A — Fundamental Ethical Principles

Item	Assessment item	Reported	Section / page	Comments / evidence
A1. Justice and equality should constitute fundamental principles in every HTA procedure.				
**A1.1**	Distribution of access to the technology across socioeconomic and geographic groups considered			
**A1.2**	Differential treatment effects across population subgroups documented			
**A1.3**	Impact on existing inequalities in care evaluated			
A2. Respect for human dignity should prevail over purely economic parameters.				
**A2.1**	Non-economic outcomes (quality of life, dignity-preserving care, patient experience) given evaluative weight independent of cost			
**A2.2**	Where cost-effectiveness alone would drive decision, ethical basis for the recommendation articulated			
A4. Non-maleficence and beneficence should be incorporated as criteria in HTA.				
**A4.1**	Potential harms (psychological burden of predictive testing, overdiagnosis, genetic discrimination) assessed alongside expected benefits			

Item	Assessment item	Reported	Section / page	Comments / evidence
**A4.2**	Benefit-harm balance evaluated for vulnerable subgroups, not only the average patient			

Domain B — Transparency, Stakeholder Participation, and Institutional Accountability

Item	Assessment item	Reported	Section / page	Comments / evidence
B1. Patient and citizen participation should be mandatory at all stages of HTA.				
**B1.1**	Patients and citizen representatives involved at the scoping, evidence appraisal, and recommendation stages			
**B1.2**	Where patient involvement was limited, structural reasons and implications for the conclusions documented			
B2. HTA procedures should be transparent and all criteria/data should be publicly disclosed.				
**B2.1**	Assessment criteria, weightings, and decision rules publicly available before the assessment is conducted			
**B2.2**	Underlying evidence sources, including any commercially confidential material, documented to the extent permissible			
B3. A statutory mechanism for objections and review of HTA decisions should exist.				
**B3.1**	Formal pathway exists for stakeholders to raise objections to the assessment outcome			
**B3.2**	Review pathway is independent of the body that issued the original decision			
B5. Professional training in HTA ethics should become a statutory requirement.				
**B5.1**	Those participating in the assessment received documented training in the ethical analysis of health technologies			
**B5.2**	Continuing professional development in HTA ethics included in the institutional capacity-building plan			

Domain C — Equity and Access

Item	Assessment item	Reported	Section / page	Comments / evidence
C1. Equal access to health services should constitute a criterion in assessments.				

Item	Assessment item	Reported	Section / page	Comments / evidence
**C1.1**	Reimbursement and delivery arrangements evaluated for equitable access across geographic and socioeconomic groups			
**C1.2**	Implementation barriers (cost-sharing, specialist availability, infrastructure) considered as part of access			
C4. Health quality indicators should be published disaggregated by social group.				
**C4.1**	Outcome data reported with disaggregation by sex, age group, socioeconomic status, and ethnic or genetic ancestry where applicable			
**C4.2**	Where disaggregated data are not available, this gap and its implications documented			

Domain D — Digital Health and Artificial Intelligence

Item	Assessment item	Reported	Section / page	Comments / evidence
D1. Digital health technologies should also be evaluated on the basis of ethical criteria.				
**D1.1**	Digital component (data flows, algorithmic decision logic, integration with clinical workflow) subject to ethical evaluation independent of the underlying clinical claim			
D2. AI technologies should be systematically checked for bias both before approval and during clinical use.				
**D2.1**	Developer documented bias evaluation across relevant population subgroups (genetic ancestry, sex, age, comorbidity)			
**D2.2**	Post-market bias monitoring required as a condition of reimbursement			
D3. The explainability of AI systems should be a prerequisite for HTA.				
**D3.1**	Clinicians using the AI system can access an explanation of how individual recommendations are generated, at a level appropriate to their decision-making role			
**D3.2**	Where full explainability is not technically achievable, compensatory safeguards (clinical override, audit trails) documented			
D4. There should always be human oversight in decisions taken with AI support.				
**D4.1**	Documented clinician-in-the-loop requirement for clinical decisions supported by AI			

Item	Assessment item	Reported	Section / page	Comments / evidence
**D4.2**	Clinicians trained and resourced to exercise meaningful oversight, not nominal sign-off			
D5. The degree of digital divide should be assessed.				
**D5.1**	Patient access to the digital infrastructure required by the technology (devices, connectivity, digital literacy) considered			
**D5.2**	Alternative pathways available for patients without that access			

Domain E — Pandemic Preparedness and System Resilience

Item	Assessment item	Reported	Section / page	Comments / evidence
E2. System readiness for supply chain disruptions or pandemics should be assessed within HTA.				
**E2.1**	Supply chain dependencies and implications of disruption for continuity of care considered			
**E2.2**	Arrangements in place for equitable allocation of the technology under crisis conditions			

Notes on use

Response categories. The four response categories are intended to capture the substantive state of the assessment, not to produce a numerical score:

Yes — The item is fully addressed in the assessment, with documented evidence.

Partial — The item is addressed but with limitations (e.g., missing data, narrow scope, unresolved methodological issue). The limitation should be explained in the comments column.

No — The item is not addressed. The reason should be documented in the comments column, especially where the item is relevant to the technology under assessment.

N/A — The item is not applicable to the technology or context (e.g., AI items for a non-AI technology). The reason should be briefly justified.

Interpretation. A predominance of Partial or No responses is not in itself a negative outcome; it indicates which dimensions of the ethical assessment require further methodological development or additional evidence collection. The checklist is therefore as much a tool for identifying gaps in the evidence base as it is for documenting compliance. Items that recur as Partial or No across multiple assessments by the same agency may indicate a systemic gap in the agency's HTA infrastructure that warrants institutional response.

Adaptation. The checklist is offered as a procedural template. Agencies adopting it should adapt the wording of items to their regulatory vocabulary and may add domain- or technology-specific items where the precision medicine application requires them. The methodological architecture (the modified Delphi process described in the main manuscript) can be replicated to generate national or sectoral variants.

Citation. If the checklist is used in published HTA reports, it should be cited as Supplementary Material S3 of the present manuscript.
