# Supplementary material for: Ethical Considerations in Health Technology Assessment for Precision Medicine: A Delphi Study in a Greek Setting
Source: J Pers Med. 2026 Jun 5;16(6):308. doi: 10.3390/jpm16060308 (PMC13301307; doi:10.3390/jpm16060308)
Supplement: Supplementary file 1 [file jpm-16-00308-s001.zip › Supplementary_Table_S1.pdf]

Supplementary Table S1. Content Validity Results for All 32 Candidate Ethical Statements (Round 1, N = 18)

Note: *Ne = number of experts rating the statement as "Necessary"; Nu = "Useful but not necessary"; Nn = "Not necessary". CVR = Content Validity Ratio (Lawshe, 1975): CVR = (Ne − N/2) / (N/2). Retention criteria: CVR ≥ 0.42 AND ≥ 80% agreement in top two response categories. Retained statements are highlighted in green; excluded statements in yellow.*

Code	Statement	Ne	Nu	Nn	CVR	% Agreement	Outcome
**Domain A: Fundamental Ethical Principles**							
**A1**	Justice and equality should constitute fundamental principles in every HTA procedure.	18	0	0	1.000	100.0%	**Retained**
**A2**	Respect for human dignity should prevail over purely economic parameters.	13	5	0	0.444	100.0%	**Retained**
A3	Patient autonomy should be taken into account in assessments.	12	6	0	0.333	100.0%	Excluded
**A4**	Non-maleficence and beneficence should be incorporated as criteria in HTA.	18	0	0	1.000	100.0%	**Retained**
**Domain B: Transparency, Stakeholder Participation & Institutional Accountability**							
**B1**	Patient and citizen participation should be mandatory at all stages of HTA.	13	5	0	0.444	100.0%	**Retained**
**B2**	HTA procedures should be transparent and all criteria/data should be publicly disclosed.	14	4	0	0.556	100.0%	**Retained**
**B3**	A statutory mechanism for objections and review of HTA decisions should exist.	14	4	0	0.556	100.0%	**Retained**
B4	Conflicts of interest of those participating in HTA should be mandatorily disclosed.	12	6	0	0.333	100.0%	Excluded
**B5**	Professional training in HTA ethics should become a statutory requirement.	13	5	0	0.444	100.0%	**Retained**
**Domain C: Equity and Access**							
**C1**	Equal access to health services should constitute a criterion in assessments.	17	1	0	0.889	100.0%	**Retained**
C2	Assessments should take into account vulnerable groups (migrants, persons with disabilities, low income).	12	6	0	0.333	100.0%	Excluded
C3	Equity indicators (e.g., language, socioeconomic status, geographic	11	6	1	0.222	94.4%	Excluded

Code	Statement	Ne	Nu	Nn	CVR	% Agreement	Outcome
	remoteness) should be systematically collected.						
**C4**	Health quality indicators should be published disaggregated by social group.	13	3	2	0.444	88.9%	**Retained**
C5	Priority should be given to younger patients over older ones in cases of limited resources.	2	7	9	-0.778	50.0%	Excluded
C6	Therapies with excessively high costs should be rejected even if they are the only option for few patients (e.g., orphan drugs).	1	6	11	-0.889	38.9%	Excluded
C7	HTA decisions should be based primarily on utilitarian approaches.	1	14	3	-0.889	83.3%	Excluded
C8	A hybrid framework (combination of utilitarianism and ethics of care) is more appropriate for HTA procedures.	10	6	2	0.111	88.9%	Excluded
C9	Personalized care and patient autonomy should take priority over utility.	5	11	2	-0.444	88.9%	Excluded
**Domain D: Digital Health and Artificial Intelligence**							
**D1**	Digital health technologies should also be evaluated on the basis of ethical criteria.	15	3	0	0.667	100.0%	**Retained**
**D2**	AI technologies should be systematically checked for bias both before approval and during clinical use.	15	3	0	0.667	100.0%	**Retained**
**D3**	The explainability of AI systems should be a prerequisite for HTA.	13	5	0	0.444	100.0%	**Retained**
**D4**	There should always be human oversight in decisions taken with AI support.	14	4	0	0.556	100.0%	**Retained**
**D5**	The degree of digital divide (e.g., access to digital infrastructure) should be assessed.	13	5	0	0.444	100.0%	**Retained**
D6	It is acceptable to adopt AI that operates as a "black box" if it is clinically effective.	3	9	6	-0.667	66.7%	Excluded
**Domain E: Pandemic Preparedness and System Resilience**							
E1	HTA should include resilience criteria for health system preparedness in public health crises.	11	6	1	0.222	94.4%	Excluded

Code	Statement	Ne	Nu	Nn	CVR	% Agreement	Outcome
**E2**	System readiness for supply chain disruptions or pandemics should be assessed within HTA.	14	4	0	0.556	100.0%	**Retained**
**Domain F: Environmental Sustainability**							
F1	The environmental footprint (carbon footprint, waste) should constitute an official HTA criterion.	7	7	4	-0.222	77.8%	Excluded
F2	Technologies with a high environmental footprint should be rejected even if clinically effective.	5	8	5	-0.444	72.2%	Excluded
F3	Healthcare procurement procedures should take into account green sustainability.	11	6	1	0.222	94.4%	Excluded
**Domain G: Social Acceptability and Public Trust**							
G1	Social acceptability of a new technology by society should be taken into account.	7	9	2	-0.222	88.9%	Excluded
G2	Public trust in the health system should be considered a quality indicator of HTA.	11	4	3	0.222	83.3%	Excluded
G3	Social impacts (e.g., isolation of elderly due to robotic care) should be evaluated on equal terms with clinical outcomes.	12	5	1	0.333	94.4%	Excluded
